# Genomic surveillance, characterization and intervention of a polymicrobial multidrug-resistant outbreak in critical care

**DOI:** 10.1099/mgen.0.000530

**Published:** 2021-02-18

**Authors:** Leah W. Roberts, Brian M. Forde, Trish Hurst, Weiping Ling, Graeme R. Nimmo, Haakon Bergh, Narelle George, Krispin Hajkowicz, John F. McNamara, Jeffrey Lipman, Budi Permana, Mark A. Schembri, David Paterson, Scott A. Beatson, Patrick N. A. Harris

**Affiliations:** ^1^​ School of Chemistry and Molecular Biosciences, University of Queensland, Brisbane, QLD, Australia; ^2^​ Australian Infectious Disease Research Centre, University of Queensland, Brisbane, QLD, Australia; ^3^​ EMBL-EBI, Wellcome Genome Campus, Hinxton, Cambridgeshire, UK; ^4^​ The University of Queensland, Faculty of Medicine, UQ Centre for Clinical Research, Brisbane, QLD, Australia; ^5^​ Infection Monitoring and Prevention Service, Royal Brisbane and Women’s Hospital, Herston, Queensland, Australia; ^6^​ Unit of Infectious Diseases, Royal Brisbane and Women’s Hospital, Herston, Queensland, Australia; ^7^​ Pathology Queensland, Central Laboratory, Brisbane, QLD, Australia; ^8^​ Nimes University Hospital, University of Montpellier, Nimes, France

**Keywords:** carbapenem resistance, *Acinetobacter baumannii*, CR-Ab, whole-genome sequencing, WGS, intensive care unit, burns ward, metagenomics, genomics, surveillance

## Abstract

**Background.** Infections caused by carbapenem-resistant *Acinetobacter baumannii* (CR-Ab) have become increasingly prevalent in clinical settings and often result in significant morbidity and mortality due to their multidrug resistance (MDR). Here we present an integrated whole-genome sequencing (WGS) response to a persistent CR-Ab outbreak in a Brisbane hospital between 2016–2018.

**Methods**. *A. baumannii, Klebsiella pneumoniae, Serratia marcescens* and *Pseudomonas aeruginosa* isolates were sequenced using the Illumina platform primarily to establish isolate relationships based on core-genome SNPs, MLST and antimicrobial resistance gene profiles. Representative isolates were selected for PacBio sequencing. Environmental metagenomic sequencing with Illumina was used to detect persistence of the outbreak strain in the hospital.

**Results.** In response to a suspected polymicrobial outbreak between May to August of 2016, 28 CR-Ab (and 21 other MDR Gram-negative bacilli) were collected from Intensive Care Unit and Burns Unit patients and sent for WGS with a 7 day turn-around time in clinical reporting. All CR-Ab were sequence type (ST)1050 (Pasteur ST2) and within 10 SNPs apart, indicative of an ongoing outbreak, and distinct from historical CR-Ab isolates from the same hospital. Possible transmission routes between patients were identified on the basis of CR-Ab and *K. pneumoniae* SNP profiles. Continued WGS surveillance between 2016 to 2018 enabled suspected outbreak cases to be refuted, but a resurgence of the outbreak CR-Ab mid-2018 in the Burns Unit prompted additional screening. Environmental metagenomic sequencing identified the hospital plumbing as a potential source. Replacement of the plumbing and routine drain maintenance resulted in rapid resolution of the secondary outbreak and significant risk reduction with no discernable transmission in the Burns Unit since.

**Conclusion.** We implemented a comprehensive WGS and metagenomics investigation that resolved a persistent CR-Ab outbreak in a critical care setting.

## Data Summary

The datasets supporting the conclusions of this article are available in the short read archive (SRA) repository, under the following Bioprojects: the complete genomes for MS14413 (GenBank: CP054302.1) and MS14393 (GenBank: CP054303-CP054305) have been deposited under the Bioprojects PRJNA631347 and PRJNA631348, respectively. All isolate Illumina sequencing reads have been deposited under the Bioproject PRJNA631491. All metagenomic Illumina sequencing reads have been deposited under the Bioproject PRJNA631351.

**Impact Statement**

Infections with carbapenem-resistant *Acinetobacter baumannii* (CR-Ab) have a high morbidity and mortality in healthcare settings and can be difficult to treat due to limited susceptibility to available antimicrobials. Rigorous surveillance and intervention methods are necessary to combat its spread within hospitals, particularly among patients in critical care. DNA sequencing has become instrumental in the detection and tracking of bacteria in hospitals, but barriers still exist to its routine implementation. Timely reporting and appropriate communication of findings are important features for the success and integration of genomics in healthcare settings. Here we present a thorough investigation of an ongoing CR-Ab outbreak in a tertiary hospital in Brisbane, Australia, between 2016–2018. Continual analysis and timely communication between the genomics and infection control teams allowed for the eradication of both the initial outbreak and recurrent infections, which were traced back to the hospital plumbing. Continued interventions have resulted in significant risk reduction to patients, with no cases in critical care since 2018. Here we provide examples of our genomics reporting scheme (both the original and the adapted version after ongoing feedback) as well as an interactive visualization of the outbreak using the Healthcare-Associated Infections Visualization Tool (HAIviz; https://haiviz.beatsonlab.com/). We believe this work to be of broad interest to both researchers and clinicians alike.

## Introduction

Hospital outbreaks of multidrug-resistant Gram-negative pathogens present great risk to patients and are costly [1, 2]. Whole-genome sequencing (WGS) has been proposed as an effective tool to support infection-control responses to emerging outbreaks within the healthcare environment, but barriers exist to the effective implementation into clinical practice [3].

*Acinetobacter baumannii* has emerged over recent decades as a major nosocomial pathogen [4]. Its capacity to develop or acquire resistance to multiple antibiotic classes, in addition to intrinsic resistance to desiccation and disinfectants, contributes to persistence of *A. baumannii* in the hospital environment [5, 6]. It has frequently been a cause of nosocomial outbreaks, particularly in the critical care setting [7–9]. *A. baumannii* are often resistant to multiple antibiotic classes and the global incidence of extensively-drug-resistant (XDR) or even pan-drug-resistant (PDR) strains have been increasing [10–12]. Carbapenem-resistant *A. baumannii* (CR-Ab) have been seen at high prevalence in several areas, particularly in the Asian-Pacific region, Latin America and the Mediterranean [13]. Carbapenem resistance in *A. baumannii* usually arises from the acquisition of genes encoding carbapenemases, particularly OXA-type carbapenemases (e.g. OXA-23), and may be associated with high mortality in vulnerable patients [14].

Here we describe a large outbreak of CR-Ab, and other co-infecting MDR Gram-negative pathogens, occurring within an Intensive Care Unit (ICU) and burns facility. Incorporation of WGS in real-time facilitated rapid characterisation of this complex polymicrobial outbreak, provided a detailed understanding of transmission pathways and helped to direct a successful infection control response.

## Methods

### Study setting and patient inclusion

Primary isolates were obtained from patients admitted to the Royal Brisbane and Women’s Hospital (RBWH), a tertiary referral hospital with 929 beds in South-East Queensland, Australia. The RBWH has a 36 bed ICU providing highly specialist burns care for all of Queensland. The incidence of CR-Ab is low in Australian hospitals [15]. All new CR-Ab strains are routinely stored in the clinical laboratory for future reference. For the outbreak investigation, any patient admitted to the RBWH who cultured CR-Ab from any clinical or screening specimen from May to August 2016 was identified as a case and included in the primary outbreak analysis. Any CR-Ab cases during the outbreak period were also included to determine if plasmid-mediated resistance and dissemination was relevant, with any MDR Gram-negative bacilli (including ESBL-producing *K. pneumoniae *, carbapenem-resistant *S. marcescens* or carbapenem-resistant *P. aeruginosa *) prospectively collected for further genomic analysis. Overall these included 28 CR-Ab, three carbapenem-sensitive *A. baumannii *, ten *K *. *pneumoniae *, seven *P *. *aeruginosa *, four *S *. *marcescens* and three *Enterobacter cloacae* (the *E. cloacae* were isolated in relation to a previous outbreak in the same hospital [16]). Stored CR-Ab isolates from a previous outbreak in 2006 [6], as well as other sporadic cases imported from overseas to the RBWH during 2015/2016 (prior to the outbreak) were included for further analysis. These included 17 historical CR-Ab isolates from earlier in 2016 (*n*=3), 2015 (*n*=2) and between 2000–2006 (*n*=12). *A. baumannii* identified from the outbreak until mid-2018 were also included in the analysis during continued surveillance and infection control monitoring. These included three carbapenem-sensitive *A. baumannii* and 19 CR-Ab isolates. A complete list of all isolates is provided in File S2 (available in the online version of this article).

### Antimicrobial susceptibility testing

All bacterial isolates were identified by MALDI-TOF (Vitek MS; bioMérieux, France). Antimicrobial susceptibility testing was carried out using Vitek 2 automated AST-N426 card (bioMérieux). For the first eight sequential CR-Ab isolates, additional susceptibility testing was undertaken using Etest to determine MICs for meropenem, imipenem, colistin, tigecycline, fosfomycin, amikacin, sulbactam, doxycyline and ceftolozane/tazobactam, with disc diffusion to determine susceptibility to aztreonam and ceftazidime/avibactam. Additional colistin testing was carried out on suspected colistin-resistant isolates using broth microdilution via the MICRONAUT MIC-Strip Colistin (Merlin Diagnostika GmbH). Carbapenemase activity was assessed by the use of the Carba-NP test (RAPIDEC; bioMérieux) and screened for the presence of common carbapenemases found in Enterobacteriaceae using an in-house multiplex real-time PCR (that targets NDM, IMP-4-like, KPC, VIM and OXA-48-like carbapenemases). Once it became clear that all the outbreak strains had an identical antibiogram, susceptibility testing was confined to the Vitek 2 automated AST-N426 panel with MICs to tigecycline, doxycycline and colistin determined by Etest (as the only susceptible agents).

### Bacterial culturing and genomic DNA extraction

All isolates were grown on horse blood agar at 37 °C overnight. For all historical and outbreak isolates collected between May–September of 2016, colonies were scraped from plates and resuspended in 5 ml Luria–Bertani (LB) broth. Then, 1.8 ml of resuspension was used for DNA extraction using the UltraClean Microbial DNA Isolation Kit (MO BIO Laboratories) as per the manufacturer’s instructions. All isolates collected after September 2016 were extracted using the DSP DNA Mini Kit on the QIAsymphony SP (Qiagen).

### Isolate whole genome sequencing

Illumina WGS of suspected outbreak patient isolates and historical CR-Ab isolates was performed in four batches of between 10 and 18 samples between June and August 2016 at the Australian Centre for Ecogenomics (ACE), The University of Queensland (see Methods in the Supplementary Material). One CR-Ab isolate (MS14413) and one *K. pneumoniae* isolate (MS14393) were selected for sequencing with Pacific Biosciences (PacBio) Single Molecule Real-Time (SMRT) sequencing on an RSII machine (see Methods in the Supplementary Material). Subsequent Illumina WGS was carried out at Queensland Forensic Scientific Services (QFSS) (see Methods in the Supplementary Material).

### Quality control and assembly of WGS data

Illumina raw reads were checked for contamination using Kraken [17] v0.10.5-beta and quality using FastQC v0.11.5 (www.bioinformatics.babraham.ac.uk/projects/). Raw reads were filtered for reads less than 80 bp and quality score less than five using Nesoni clip v0.130 (https://github.com/Victorian-Bioinformatics-Consortium/nesoni). Some reads required further hard trimming with Nesoni clip (10 bp from start, 40 bp from end). Isolates were assembled using SPAdes [18] v3.6.0 at default settings. Contigs less than 10× coverage were removed using a custom script. Assembly metrics were checked for quality using Quast [19] v4.3 (see File S2). Details of the PacBio genome assembly and annotation can be found in Methods in the Supplementary Material (Fig. S1, Table S1).

### Genomic analysis and clinical reporting

Between June and August 2016, four reports of detailed bioinformatic analyses were prepared in response to available Illumina data for *A. baumannii *, *K. pneumoniae *, *P. aeruginosa *, *S. marcescens* and *Enterobacter cloacae* patient isolates. Comparative genome analysis using variant calling, phylogenetic reconstruction, transmission pathway prediction, MLST resistance gene prediction and plasmid characterization used in the clinical reports are given in Methods in the Supplementary Material. For subsequent analyses of the final genome dataset updated or alternative software was used as described below.

Core SNPs were identified using Snippy [20] (v4.3.6) at default settings and trimmed reads against the complete chromosomes for MS14413 (CR-Ab) and MS14393 (* K. pneumoniae *). Parsnp (v1.2) (at default with ‘-c’ flag) was used to visualize phylogenetic relatedness between the outbreak CR-Ab and the historical *A. baumannii* isolates. MLST was performed using mlst [21] v2.6 (https://github.com/tseemann/mlst) against the draft assemblies. Both the Oxford [22] and Pasteur [23] MLST schemes were used for the CR-Ab isolates. Resistance genes were identified using Abricate [24] v0.6 against the ResFinder database [25] (accessed 18 August 2017). Abricate was also used to determine plasmid types using the PlasmidFinder database [26] (accessed 18 August 2017). Comparative analyses were completed using the Artemis Genome browser and the Artemic Comparison Tool (ACT). Figures were constructed using EasyFig [27], BRIG [28] and FigTree [29]. The capsular polysaccharide (K) and lipooligosaccharide outer core (OCL) locus for *A. baumannii* were typed using Kaptive v0.5.1 [30] against the *A. baumannii* databases provided [31] (accessed 16 Dec 2020).

### Metagenomic sequencing and analysis

Metagenomic sequencing of environmental samples and analysis was conducted as described previously [16]. Briefly, swab and water samples from the ICU and Burns Unit were collected in July 2018. DNA was extracted using the Qiagen DNeasy Powersoil extraction kit and sequenced at the Australian Centre for Ecogenomics on an Illumina NextSeq500. Metagenomic sequencing data was used to screen for evidence of the current *A. baumannii* outbreak strain, as well as a previously identified *Enterobacter hormaechei* strain responsible for an outbreak in the same ICU in 2015 (described in [16]).

All samples were screened for species using Kraken [17] v1.0 and resistance genes using SRST2 [32] v0.2.0 against the ARG-ANNOT [33] database. Mash [34] v1.1.1 was used at default settings to screen Illumina reads for each samples against our reference CR-Ab sketch (MS14413). Samples that shared ≥90% of hashes were mapped to the reference sequence. Mapped reads were parsed and *de novo* assembled using SPAdes [18] v3.11.1 for MLST analysis using mlst [21] v2.16.2 and nucleotide comparison using ACT [35] and BRIG [28].

### Risk reduction assessment

We aimed to estimate the reduced risk of patient colonization following the identification of ST1050 CR-Ab by environmental metagenomic sequencing and the initiation of enhanced decontamination of hospital plumbing. The incidence rate of CR-Ab was measured pre-intervention and post-intervention. The point of intervention was defined as the targeted initiation of routine plumbing maintenance programme within the Burns and Intensive Care units in August 2018. The intervention was expected to generate immediate results with no lag time. The pre-intervention period was defined as May 2016 to August 2018 and post-intervention period as September 2018 to May 2020. All CR-Ab cases recorded in the hospital during these periods were included. Patients admitted to the Burns and Intensive Care units underwent standard clinical swabbing for surveillance and laboratory method for testing did not change over the study period. Statistical analyses were performed on Rv3.5.1.

## Results

### Case study

A 25-year-old patient with extensive burn injuries was retrieved from an overseas healthcare facility. As per infection-control protocols, the patient was placed on contact precautions and provided a single room. Initial nasal and rectal screening swabs were negative for MDR pathogens, including CR-Ab. An extended-spectrum beta-lactamase (ESBL)-producing *Klebsiella pneumoniae* was isolated from the patient’s respiratory secretions on day 4, and within 24 h a similar organism was isolated from blood cultures. Repeated collection of blood cultures demonstrated a polymicrobial culture with ESBL-producing *K. pneumoniae *, CR-Ab and *Pseudomonas aeruginosa* on day 6, that tested susceptible to all first-line agents. Over the following days, CR-Ab was also isolated from numerous clinical specimens, including a femoral line tip, endotracheal aspirates, rectal swabs, wound swabs and operative specimens collected from debrided tissue. Blood cultures repeatedly grew CR-Ab, (day 15 and 45 of admission), with the emergence of colistin resistance when tested by Etest (MIC 32 μg ml^−1^) on day 45. *Serratia marcescens* was co-cultured in blood on day 15 and was also grown from respiratory secretions and wounds swabs.

Over the next 5 months in 2016, 18 additional patients within the same ICU area were also found to be colonized or infected with phenotypically similar CR-Ab, *K. pneumoniae *, *S. marcescens* and/or *P. aeruginosa *. This included CR-Ab colonized cases identified in patients discharged from the ICU to the Burns Unit or other surgical wards throughout the hospital, and eventually patients admitted to the Burns Unit. The final CR-Ab case was identified several weeks later in a patient discharged from the Burns Unit and transferred to a hospital in a remote part of Queensland. An outbreak investigation team was constituted as soon as it was suspected that an outbreak of CR-Ab had occurred within the ICU and the use of WGS for strain characterization was initiated.

### WGS predicted likely transmission pathways and ruled out non-outbreak cases

Between May to August 2016, a total of 55 isolates were recovered from 22 patients (see File S2). These isolates included *A. baumannii *, *K. pneumoniae *, *S. marcescens, E. cloacae* and *P. aeruginosa *. Species typing and antibiogram analysis alone were insufficient to determine clonal relationships between these isolates. As such, we used WGS to establish the relationship between isolates and predict patient transmission based on SNP accumulation.

We applied WGS in real-time over the course of the outbreak. Four reports aimed at communicating genomic analyses to infection control and other clinical staff at RBWH were delivered during the primary outbreak (22 June, 15 July, 2 August and 29 August). We managed on average a 1 week turn-around time between receiving the isolates and presenting a finalized report, which consisted of (i) a front-page overview of the analysis and key outcomes/interpretations conveyed as short bullet points, (ii) detailed analysis and diagrams on the internal pages, and (iii) method descriptions (see Methods in Supplementary Material). Actual time between receipt of sequencing data and reporting was 8–72 h depending on the complexity of analyses with supplementary interim reports and regular academic-clinical partner meetings necessary to communicate our comparative genomic analyses and help shape the content of the final reports (see File S1 for example reports from 22 June and 29 August, respectively). The Hospital-Acquired Infections Visualization tool (HAIviz) was also developed alongside analysis of this outbreak and was used to interactively display linked cases throughout the hospital wards [File S3 (video)].

**Fig. 1.**
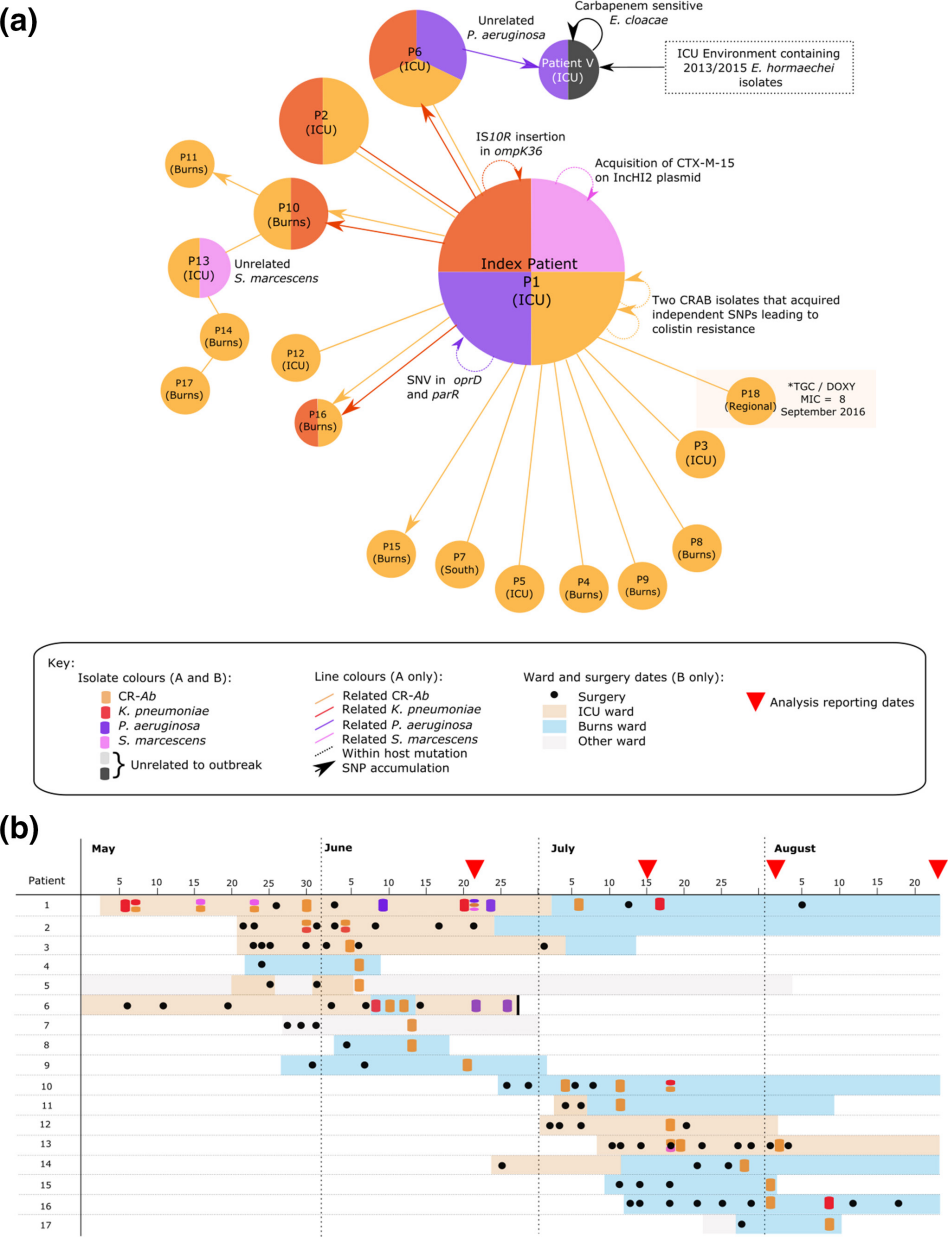
Patient relationship matrix describing 2016 outbreak of CR-Ab: (a) Each circle represents a patient, where the size of the circle correlates to the number of isolates from that patient. Colours correspond to bacterial species. Straight lines connecting circles represent patients with identical isolates (with the colour of the line indicating the specific species) at the core-genome level (and as such directionality of transmission cannot be inferred). Lines with arrows (also coloured by species) represent predicted direction of transmission based on the accumulation of SNPs between patients’ isolates. Circular arrows represent changes in individual patient’s isolates, (b) timeline of patient samples, as well as location and surgery dates.

The presumed index patient admitted in early May 2016 was identified with ST1050 (Pasteur ST2) CR-Ab, ST515 *K. pneumoniae *, ST979 *P. aeruginosa* and *S. marcescens *. Using WGS, we found that 16 of the 21 patients admitted following the index patient had bacterial infections related to either the ST1050 CR-Ab or the ST515 *K. pneumoniae *. Transmission direction based on the accumulation of SNPs was inferred in patients 10, 11, 13, 14, 15, 16 and 17 (Fig. 1a, as indicated by lines with arrows). CR-Ab isolates from the first nine patients (and patient 12) were identical based on core SNPs, making inference of patient transmission impossible using SNPs alone. However, when combined with SNP information from *K. pneumoniae* isolates, it was possible to infer co-transmission of *K. pneumoniae* and CR-Ab from the index patient to patient 6 (Fig. 1).

Strains of *S. marcescens* and *P. aeruginosa* specific to the index patient were not found in other patients (see Results in Supplementary Material). Two patients had unrelated *S. marcescens* and *P. aeruginosa* isolates (patients 13 and 6, respectively). Transmission of the unrelated *P. aeruginosa* isolate from patient 6 to another patient in the ICU ward (denoted patient V) was detected. Patient V was also found to have an *Enterobacter cloacae* isolate (later identified as *Enterobacter hormaechei* by WGS) identical to that identified in a 2015 outbreak from the same hospital [16]. This patient also carried an additional carbapenem-sensitive *E. cloacae* (*bla*IMP-4 negative) that was unrelated to the carbapenem-resistant isolate.

Over the course of the outbreak, each species carried by the index patient acquired additional antibiotic resistance mechanisms, via mutations (*pmrB* mutations in CR-Ab*,* IS*10R* insertions in *ompK36* in *K. pneumoniae, oprD* nonsense mutation in *P. aeruginosa *) or plasmid gain (IncHI2 in *S. marcescens *) (Fig. 1, Results in Supplementary Material and Tables S2 and S3).

An additional CR-Ab was isolated in September 2016 from a patient in a Regional Queensland (QLD) hospital who had previously been admitted to the Brisbane ICU (patient 18, isolate MS14438). Analysis of this isolate found that it was closely related to isolates from the initial outbreak between May to August 2016.

Extensive environmental swabbing throughout the ICU and Burns Unit was conducted on the 16 June 2016, targeting patient bedrooms as well as high-touch areas (e.g. nurse keyboards, trolleys, door handles). However, no bacterial species related to the CR-Ab outbreak were detected in the environment based on traditional culture methods using chromogenic agar.

### The outbreak CR-Ab was likely imported into the hospital ICU

In total, 29 CR-Ab isolates related to the ongoing outbreak were collected from 18 patients between May–September 2016. All were found to be ST1050 (Pasteur ST2; global clone [GC] 2) and less than ten SNPs different (Fig. S2). Three carbapenem-sensitive *A. baumannii* isolated at the same time were found to be different sequence types and unrelated to the outbreak. Comparison of the outbreak ST1050 CR-Ab isolates to historical CR-Ab isolates collected between 2000–2016 from the hospital found no close relationship, indicating that the CR-Ab had likely been introduced into the hospital with the index patient (Fig. S3).

All ST1050 CR-Ab isolates related to the index were found to be extensively resistant to carbapenems, β-lactams, cephalosporins, aminoglycosides and quinolones (Table 1). Resistance to colistin appeared in three isolates from the index patient and was mediated by two independent SNP acquisitions in the sensor kinase gene *pmrB* (causing the amino acid changes T235I in MS14413 and its descendant MS14402, and R263C in MS14407). These SNP corresponded to MICs of 8, 16 and 64 µg ml^−1^ for MS14402, MS14413 and MS14407, respectively, by broth microdilution. Antibiotic resistance genes were conserved between all isolates, and included β-lactamases (such as *bla*_OXA-23_ and *bla*_OXA-66_), streptomycin resistance genes (*strA* and *strB*), and aminoglycoside resistance genes [*aph(3′)-Ic*, *aadA1* and the methylase *armA*]. Finally, a single SNP was found to result in the reversion of a nonsense mutation in a putative type 3 filamentous fimbriae gene (*filB*). This SNP was identified in the majority of CR-Ab isolates taken after the 4 July 2016 and appears to have arisen independently multiple times across the *A. baumannii* lineage (Results in Supplementary Material, Fig. S4).

**Table 1.** CR-Ab MICs and AB resistance genes: table only shows select representative isolates as all CR-Ab were found to have the same AB resistance gene profile and MIC data. Colours represent mechanism of detection: blue, Etest MIC; Green, Disk diffusion zone diameter; Orange, Vitek2; Grey, Resfinder (accessed August 2017). *A. baumannii* is intrinsically resistant to penicillin and cephalosporins [50]

Strain		MS8413	MS8419	MS8436	MS8442	MS8441
Patient		4	5	6	7	8
Site		Leg wound	ETA	Tissue buttock	Wound Swab	Rectal Swab
Colistin	Colistin	0.125	0.25	0.25	0.125	0.5
Carbapenem	Mero	32	>32	>32	>32	>32
	Imi	>32	>32	n.t	>32	n.t
	Erta	>32	>32	>32	>32	>32
Beta-lactam and Cephalosporins	Sulb	32	32	64	32	64
	MER	R	R	R	R	R
	TIM	R	R	R	R	R
	TAZ	R	R	R	R	R
	CRO	R	R	R	R	R
	CAZ	R	R	R	R	R
	FEP	R	R	R	R	R
	KZ	R	R	R	R	R
	Azt	6 mm R	6 mm R	n.t	6 mm R	n.t
	CTZ/TAZ	>256	>256	128	16	96
	CAZ/AVI	16 mm R	17 mm R	18 mm R	15 mm R	18 mm R
	blaADC-25	+	+	+	+	+
	blaOXA-23	+	+	+	+	+
	blaOXA-66	+	+	+	+	+
Aminoglycosides	Amikacin	>256	>256	>256	>256	>256
	GENT	R	R	R	R	R
	TOB	R	R	R	R	R
	Aph(3′)-Ic-1	+	+	+	+	+
	aadA1	+	+	+	+	+
	armA	+	+	+	+	+
Quinolones	CIP	R	R	R	R	R
	NOR	R	R	R	R	R
Trimmethoprim/ Sulphonamide	TMP	R	R	R	R	R
	SXT	R	R	R	R	R
	Sul1	+	+	+	+	+
	Sul2	+	+	+	+	+
Tigecycline	Tige	2	2	2	2	4
Chloramphenicol	Chloro	6 mm R	6 mm R	n.t	6 mm R	n.t
	catB8	+	+	+	+	+
Fosfomycin	Fosfo	256	512	n.t	128	n.t
Tetracycline	Doxy	4	4	2	2	2
Macrolides	mph(E)_3	+	+	+	+	+
	msr(E)_4	+	+	+	+	+
Streptomycin	strA	+	+	+	+	+
	strB	+	+	+	+	+

n.t, not tested; Mero, Meropenem; Tige, Tigecycline; Sulb, Sulbactam; CTZ/TAZ, Ceftolozane/tazobactam; CAZ/AVI, Ceftazidime/avibactam; Chloro, Chloramphenicol; Fosfo, Fosfomycin; Azt, Aztreonam; Erta, Ertapenem; Doxy, Doxycycline; Imi, Imipenem; KZ, Cephazolin; TMP, Trimethoprim; SXT, Co-trimoxazole; GENT, Gentamicin; TOB, Tobramycin; CRO, Ceftriaxone; CAZ, Ceftazidime; FEP, Cefepime; TAZ, Piperacillin/tazobactam; CIP, Ciprofloxacin; NOR, Norfloxacin; MER, Meropenem; TIM, Ticarcillin/clavulanate.

### PacBio sequencing of CR-Ab reveals context of resistance genes and mobile elements

Complete sequencing of a reference CR-Ab isolate (MS14413) from the index patient using long-read sequencing provided a high-quality reference and allowed contextualization of the antibiotic resistance genes (as well as other mobile genetic elements) within the genome. Assembly of the ST1050 CR-Ab reference genome revealed a 4 082 498 bp chromosome with no plasmids. *StrA, strB* and *sul2* resided within a novel AbGRI1 resistance island most closely related to the *A. baumannii* strain CBA7 (GenBank:NZ_CP020586.1) isolated from Korea in 2017, both of which lacked the *tetA-tetR* genes commonly found in AbGRI1 (Fig. S5). The CR-Ab isolates also carried Tn6279 (also known as AbGRI3-2), which encompassed a large number of resistance genes including *mph(E*) and *msr(E*) (macrolide resistance) and the methylase gene *armA* (gentamicin resistance) (Fig. S6). Resistance to carbapenems in these CR-Ab isolates was likely driven by the presence of three copies of *bla*_OXA-23_ residing in separate Tn2006 transposons within the chromosome (two copies proximal to the capsule region, and the third interrupting a diguanylate cyclase gene, which has previously been implicated in biofilm formation [36]). An IS*Aba1* insertion sequence upstream of the chromosomal *ampC* gene was also detected, which has previously been shown to enhance cephalosporin resistance [37]. Additionally, an IS*Aba125* element was identified upstream of the *csu* operon, which is a well-characterized chaperone-usher pili assembly system involved in biofilm formation [38].

**Fig. 2.**
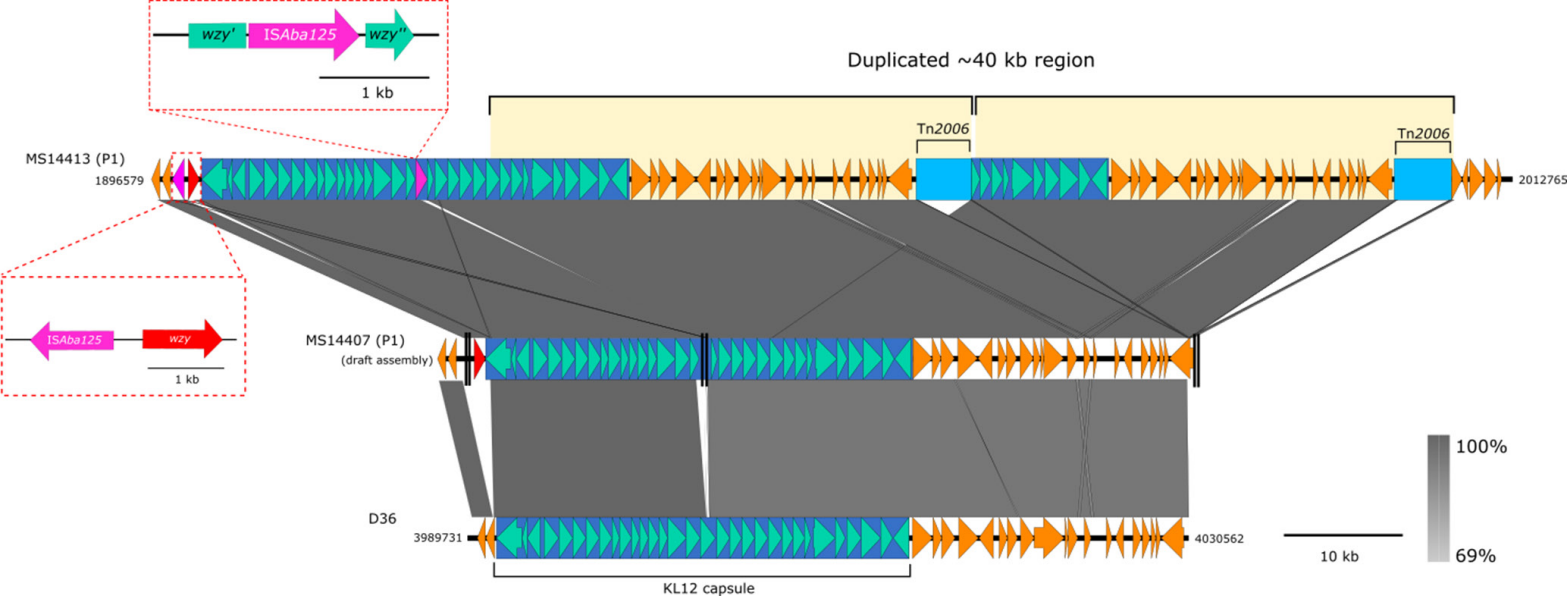
Large ~40 kb tandem duplication found in MS14413: Duplication of part of the capsule (k) region in the MS14413 complete genome (top line), resulting in three chromosomal copies of Tn*2006* (third copy at alternate locus). This duplication appears to have arisen in some of the index patient isolates, but not other isolates involved in the outbreak (e.g. MS14407 concatenated draft genome, central line; vertical double black lines represent contig break in the draft assembly, presumed to be caused by the same IS as in MS14413). The *wzy* gene in the capsule region was found to be interrupted by an IS*Aba125* element, however a secondary *wzy* gene was identified at the start of the capsule region. Neither the IS*Aba125* insertion or secondary *wzy* gene is found in the KL12 capsule locus of *A. baumannii* strain D36 (bottom line).

Long-read sequencing revealed an OCL1 oligosaccharide outer core and a KL12 capsule (K) locus, which shares 97% nucleotide identity to the capsule region found in the GC1 *A. baumannii* strain D36 (GenBank:NZ_CP012952.1) (Fig. S7). However, the *wzy* gene (a polymerase required for capsular polysaccharide biosynthesis) within the capsule locus was interrupted by an IS*Aba125* insertion sequence in all CR-Ab isolates. Further comparative analysis found a portion of the capsule locus in MS14413 to share 99% nucleotide identity to the capsule from *A. baumannii* strain BAL_097 (GenBank: KX712116), which carries a *wzy* gene at the beginning of the capsule region. This unusual gene placement also appears in MS14413, and likely complements the loss of the internal *wzy* gene (Fig. 2). The high nucleotide identity at this region also indicates possible recombination.

Overlapping the capsule (K) region in MS14413 is a large 41 375 kb tandem duplication, encompassing two copies of Tn*2006* (Fig. 2). Analysis of the other CR-Ab isolates using the Illumina *de novo* assemblies found evidence for this duplication in only one other related colistin-resistant isolates from the index patient (MS14402), suggesting that this duplication arose once and was maintained by a sub-population of CR-Ab within this patient for at least 36 days.

### Transmission of *K. pneumoniae* parallel to CR-Ab transmission

Ten ESBL-producing *K. pneumoniae* isolates were collected from five patients during the outbreak and were all found to be ST515. Nine of the ten isolates differed by less than ten core SNPs, indicating direct transmission within the ICU ward (Fig. S8). A single isolate from the index patient (MS14418) was found to have an additional 61 core SNPs, consistent with a hypermutator phenotype. Further investigation of this isolate found an in-frame 9 bp deletion in *mutH*, resulting in the loss of 3 amino acids from this protein (Fig. S9).

All ESBL-positive *K. pneumoniae* isolates had identical antibiotic resistance gene profiles, including the ESBL gene *bla*_CTX-M-15_, other β-lactamases (*bla*_TEM_, *bla*_OXA-1_) and the aminoglycoside resistance gene *aac(6′)Ib-cr* (Fig. S6). Two isolates from the index patient (MS14393 and MS14418) developed resistance to carbapenems, which was likely due to an IS*10R* insertion in the outer membrane porin gene *ompK36* (Fig. S8). Isolate MS14433 (from patient 16) also contained an IS*10R* inserted into *ompK36*, however the insertion was found to be close to the 5′ boundary of the *ompK36* gene and based on *in silico* analysis there was no evidence that it affected the function of the resulting protein. Isolate MS14393 (from the index patient) also possessed a nonsense mutation in the antibiotic resistance protein repressor gene *marR*, which could contribute to its overall resistance to antibiotics.

A single *K. pneumoniae* isolate from the index patient (MS14393) was sequenced using PacBio long-read sequencing to generate a high-quality reference genome, consisting of a 5 492 431 bp chromosome, a 216 803 bp IncF plasmid (pMS14393A), and a 125 232 bp IncA/C plasmid (pMS14393B). Most of the antibiotic resistance genes resided on the IncA/C plasmid in two main loci (Fig. S6). The larger IncF plasmid did not contain any antibiotic resistance genes, but did harbour several heavy metal resistance operons, including resistance to copper, arsenic and mercury (Fig. S10). Comparison of the short-read assemblies to both plasmids confirmed that all ten *K *. *pneumoniae* isolates retained both plasmids.

### Whole-genome shotgun metagenomics detects CR-Ab in hospital environment

**Fig. 3.**
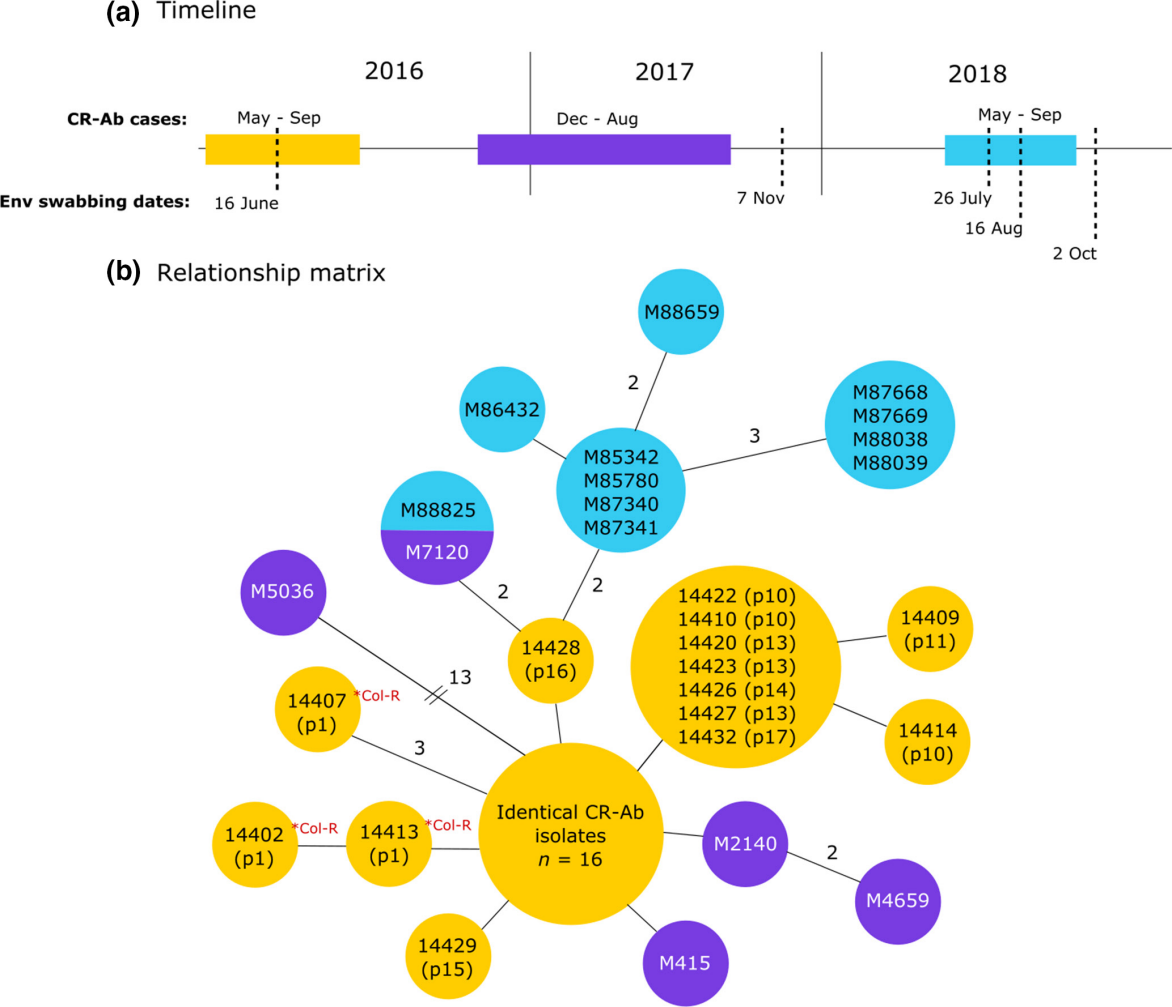
Ongoing CR-*Ab* surveillance from 2016 to 2018: (a) timeline of CR-Ab cases and dates of environmental swabbing between 2016–2018. (b) Relationship matrix of all CR-Ab isolates related to the initial outbreak. Col-R=predicted colistin resistance via mutation in *pmrB*. Isolates within the same circle are identical at the core genome. Branches represent 1 SNP difference (except where specified). Isolates from the original 2016 outbreak are in yellow. Purple isolates were collected in late 2016–2017. Isolates in blue were collected in 2018. Isolate M88825 was isolated from an Antechamber environment in 2018 and found to be identical at the core SNP level to M7120, isolated in August 2017.

Ongoing surveillance was conducted using WGS following the initial outbreak. Despite continual environmental cleaning and routine swabbing, the outbreak CR-Ab strain persisted through to September 2018 (Fig. 3). Swabs collected from surfaces within the ICU and Burns Unit (e.g. handles, tables, shelves, computer equipment) in 2016 and 2017 were unable to detect CR-Ab in the environment and did not yield enough DNA for direct metagenomic sequencing (data not shown).

**Fig. 4.**
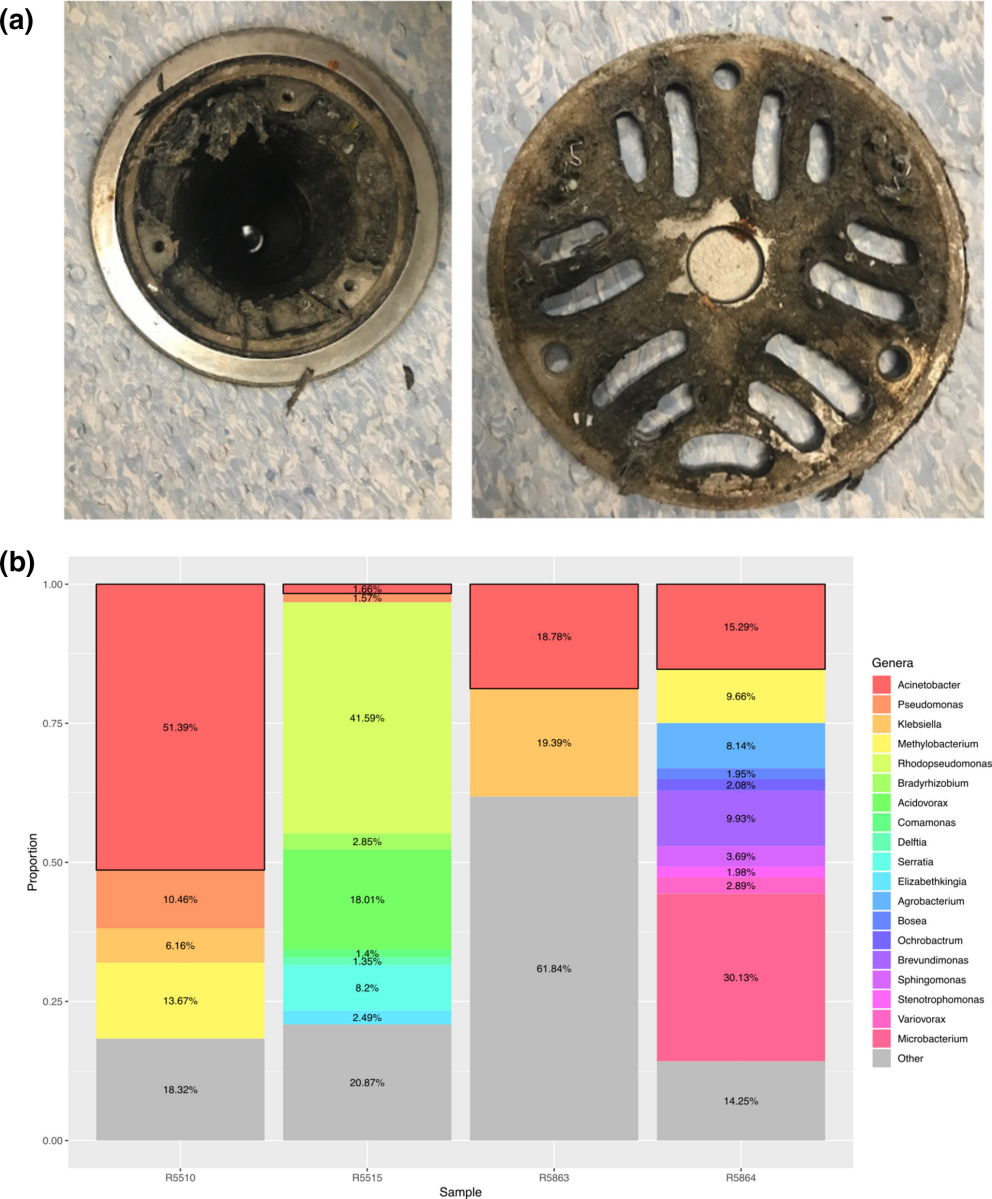
Burns bath 3 floor trap and metagenomic read abundance profiles: (a) an example of the biomass uncovered under the floor trap in a Burns Unit bathroom. Areas of high biomass (such as this one) were targeted for environmental screening. (b) Each column shows the relative abundance of paired-end reads for each environmental sample that were classified at a bacterial genus level by comparing against a database of bacterial genomes from RefSeq. Only bacterial genera with a relative abundance >0.5% are shown as distinct. Genera with an abundance of <0.5% are grouped together as ‘Other’ (grey). Boxes outlined in black represent abundance of ‘* Acinetobacter *’.

Due to 11 new cases of CR-Ab detected between May to September 2018, additional environmental sampling was carried out in the Burns ward environment. Between July to October 2018, areas of presumed high bacterial load (such as floor drains, plumbing, inside burns bath drains etc) were targeted for environmental sampling (Fig. 4). All samples were subjected to culture using traditional methods (on chromogenic media) and direct DNA extraction and shotgun metagenomic sequencing. Of 50 environmental samples, two were culture positive for CR-Ab (R5666 and R5864), while four were positive based on analysis of the metagenomic sequencing data (R5515, R5510, R5863 and R5864) (Table S4, Fig. 4).

**Fig. 5.**
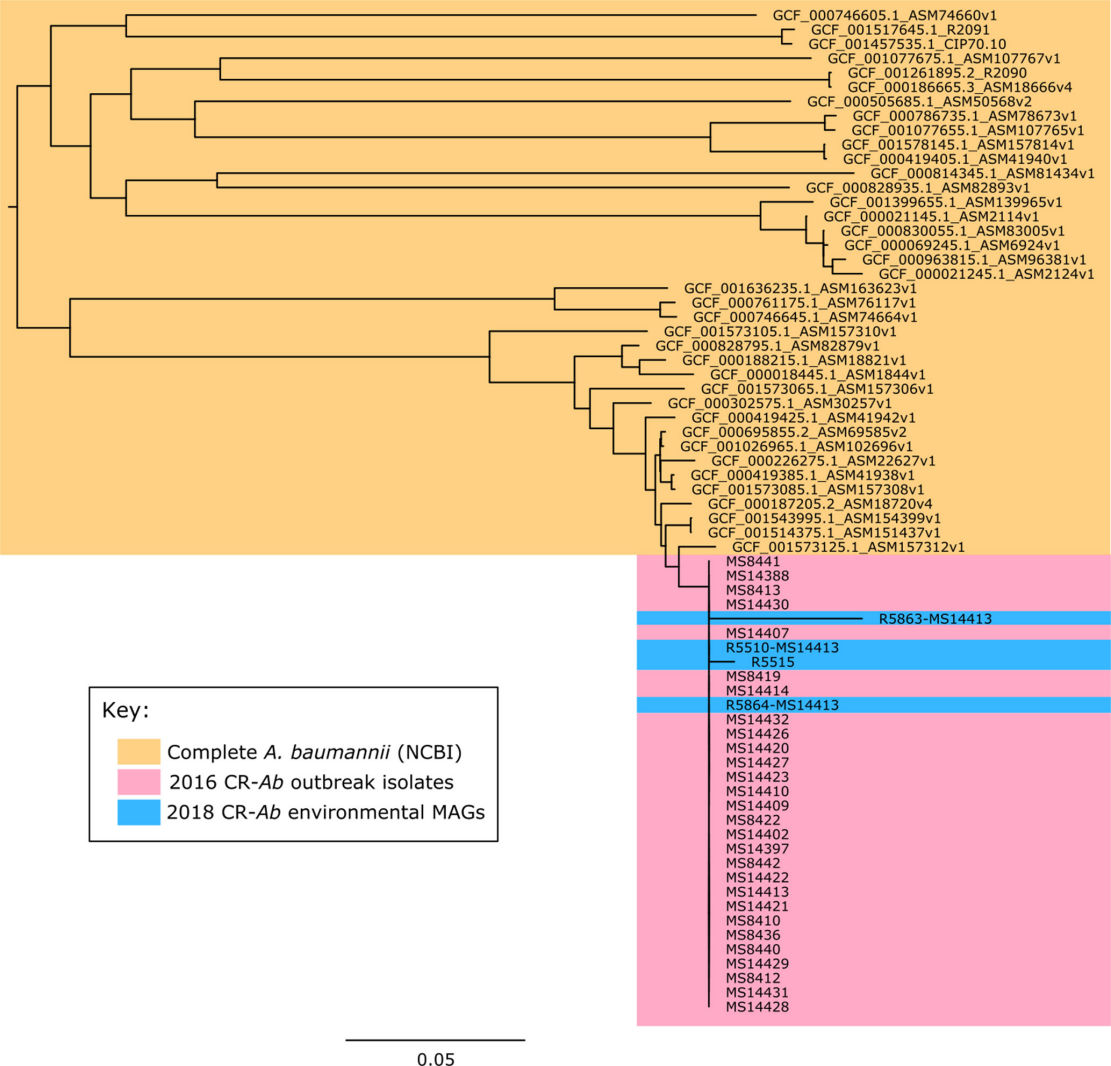
Clustering of MAGs with outbreak strains: Mid-point rooted core genome SNP phylogenetic tree contextualizing the metagenome assembled genomes (MAGs) with *de novo* assemblies of the outbreak strains and publicly available complete *A. baumannii* genomes (yellow) showing clustering of the MAGs (blue) within the outbreak clade (pink).

An ST1050 CR-Ab was cultured using traditional methods from the environmental sample R5666, taken from a crack in a toilet seat being used by a patient colonized with the ST1050 CR-Ab. The depth of sequencing obtained from the same environmental sample, however, was not sensitive enough to be able to confidently detect the presence of the CR-Ab in the metagenomic data. The second positive ST1050 CR-Ab culture came from an environmental sample taken from an Antechamber room connected to patient rooms that had previously been colonized with ST1050 CR-Ab (R5864). Parallel metagenomic sequencing was also able to detect this same ST1050 CR-Ab in the environmental sample (Fig. 5).

Additionally, three other samples were found to have ST1050 CR-Ab based on metagenomic sequencing, despite being culture negative using traditional methods (Fig. 5, Table S4). Samples R5515 (burns bath 2 floor trap water sample) and R5510 (burns bath 2 bath drain hole [interior]) were both positive for ST1050 CR-Ab. Both samples were taken at the same time from proximal locations, and patients colonized with ST1050 CR-Ab were using the burns bath in question. Samples R5863 was also positive for ST1050 CR-Ab, and was taken from the room previously occupied by a patient known to be colonized with ST1050 CR-Ab.

### Plumbing maintenance programme implemented in response to genomic investigation

Shotgun metagenomic detection of the outbreak strain in the hospital plumbing provided the evidence base for implementation of a sustainable infection prevention strategy. Consequently, a routine plumbing maintenance programme was instituted. Every month, pipes were soaked for 30 min in sodium hydroxide, with additional soaking and scrubbing of drain plates. Since the implementation of these measures, no further cases of CR-Ab have been detected in the Burns Unit or Intensive Care Unit (ICU) following 28 September 2018. Periodic environmental surveillance of CR-Ab in drains and plumbing in the Burns unit has been ongoing as of May 2020.

### Significant reduction of risk following interventions

A total of 32 CR-Ab cases were recorded over 28 months in the pre-intervention period, compared to four CR-Ab cases over 21 months in the post-intervention period. All cases identified at pre-intervention period were admitted to the Burns or Intensive Care Units during their hospitalization. Conversely, three out of the four CR-Ab cases detected in the post-intervention period had no obvious epidemiological link to exposure in the Burns and Intensive Care Units. The fourth case attended an external wound clinic, which was also attended by RBWH patients, although no specific patient or environmental link was proven. The last CR-Ab case detected in the Burns and Intensive Care unit was in September 2018, and there was no new case detected in the hospital after May 2019 (Fig. S11). The incidence rate post-intervention was two CR-Ab cases per year, significantly reduced from the pre-intervention incidence rate of 13 CR-Ab case per year (*P*<0.001). The post-intervention CR-Ab incidence rate was reduced by 17 % compared to the pre-intervention period (incidence rate reduction=0.17, 95 % CI: 0.06–0.47).

## Discussion

CR-Ab are an increasingly dire threat to global public health. Their proficiency at surviving for long periods of time in environments whilst under antibiotic pressure is largely due to the positive selection of both intrinsic and acquired resistance and survival mechanisms. As such, they present a significant problem in healthcare settings, which typically have high antibiotic use as well as a large cohort of vulnerable patients. Understanding the mechanisms behind their resistance and transmission, as well as their possible environmental reservoirs, is key to combating further colonization and infection in hospital settings. Here we present a comprehensive analysis of an outbreak of CR-Ab using isolate and environmental metagenomic sequencing to fully elucidate transmission, determine new cases rapidly and detect possible environmental reservoirs within the hospital.

Genomics is being rapidly established in clinical settings, particularly in response to outbreaks [39, 40]. This is due not only to the higher discriminatory power that WGS provides, but also the complete picture that WGS captures by yielding the entire genome. The current cost and turnaround time for sequencing and analysis also make this type of investigation more feasible in nosocomial settings. In this study, initial sequencing of the outbreak CR-Ab isolates (and associated bacterial species) confirmed an already suspected outbreak, and so despite providing more insight into possible transmission routes, it did not greatly affect the infection control response. However, genomics superseded traditional methods when it came to (i) contextualizing outbreak isolates with previous CR-Ab strains from the hospital (to determine the likely source), and (ii) contextualizing new CR-Ab isolates as they appeared after the initial outbreak to determine whether there was an ongoing problem in the hospital. While having a slightly faster turnaround time, traditional methods alone would not have been able to confidently assess either of these scenarios. Regular meetings and reporting of the genomic results provided the hospital with actionable information and greater insight into the ongoing outbreak. These cross-disciplinary discussions facilitated the communication of complex genomic data into the clinical setting, providing guiding principles for subsequent WGS reporting of multidrug resistant bacterial pathogens at this hospital, and prompting the development of an interactive online visualization for communicating genomic epidemiology data (see HAIviz; File S3).

In addition to providing evidence for related isolates, WGS was also a valuable tool for discerning unrelated isolates, in many cases preventing ward or operating theatre closures and mitigating the associated financial costs to the hospital [41, 42]. It is plausible that with continued, ongoing sequencing of clinically significant bacteria in high-risk environments (e.g. ICU and Burns Unit) the risk of outbreaks could be reduced if evidence of transmission was detected early. During this study, we were able to detect transmission of an *E. hormaechei* unrelated to the outbreak at hand, but linked to a *bla*IMP-4 carbapenemase-producing Enterobacteriaceae (CPE) outbreak from the same hospital the year prior [16]. We were also able to identify transmission of an unrelated meropenem-resistant *P. aeruginosa* isolate, highlighting how WGS can detect transmission well before it becomes known to staff. Routine WGS can also lead to a reduction in the costs associated with responding to an established outbreak. A study of a similar outbreak in Brisbane determined the cost per patient related to the outbreak to be six-times higher than unrelated patients [43]. However, the feasibility (i.e. access to sequencing facilities and analysis) of routinely sequencing multidrug-resistant organisms is not yet achievable for many hospitals, particularly in low-resource settings. Despite this, recent collaborative efforts in the Philippines [44] have demonstrated how retrospective sequencing and capacity building for prospective sequencing can be achieved. WGS is also becoming increasingly cheap and portable (e.g. Oxford Nanopore Technology), and when coupled with more accessible cloud-based infrastructure, routine sequencing in these settings has greater potential.

Determining relatedness and transmission using genomics has historically relied on the number of core SNP differences between isolates [45–47]. However, this approach has several flaws, including a general lack of consensus on SNP cutoffs and what number defines a related isolate within a particular species, as well as the fact that it largely ignores other genomic differences, such as large insertions, inversion and rearrangements. It also does not account for hypermutators, which we observed in the case of the *K. pneumoniae* isolate MS14418. More recent methods have explored the use of transmission probabilities by taking into account isolation time and species mutation rate [48], but these methods appear more suited to outbreaks spanning large timeframes. Most studies to date that have used SNP distances have used them retrospectively and under research conditions, thereby avoiding the necessity to conform to standardized metrics and allow case-by-case judgments to be made on isolates. Moving forward, translating this approach into standardized clinical settings will likely present several hurdles. In our study, with the exception of the hypermutator strain MS14418 there was no ambiguity using SNP distances to determine relatedness due to the observed low mutation rate. However, because of this, many isolates were unable to be discriminated, with several identical at the core-genome level. We were surprised that the initial polymicrobial nature of this outbreak enabled deduction of transmission routes by examining SNP differences between their respective companion *K. pneumoniae* isolates, which appeared to have coinfected with the CR-Ab. However, all of these transmissions were from the index patient and were already recognized by the clinical team. In contrast, the spread of CR-Ab between the ICU and Burns Units in July could be traced to transmission of CR-Ab carrying a discriminatory SNP from the index patient to patient 10 in the Burns Unit with subsequent transmission of CR-Ab to patient 11, 14 and 17 in the Burns Unit and patient 13 in the ICU (Fig. 1). Further work into routinely automating the identification of both SNPs and pan-genome markers (such as gain/loss of regions or movement of mobile elements) could assist in further characterizing this outbreak and others.

Metagenomic sequencing of the environment was able to identify several areas positive for ST1050 CR-Ab. In one case, metagenomic sequencing analysis and traditional culture methods were concordant and both identified the ST1050 CR-Ab. In all other cases, either traditional culture or metagenomic sequencing was able to recover the ST1050 CR-Ab, highlighting the advantage of using both methods during an outbreak. In addition to the ST1050 CR-Ab, we were also able to use the metagenomic sequencing data to search for a previously identified *E. hormaechei* strain, which caused a small outbreak in 2015 in the same ICU [16] and was found again during this study. This highlights the wider utility of metagenomic sequencing to search for not only a single strain of interest, but potentially several, while also gaining greater insight into the microbial communities within the hospital plumbing.

While metagenomic sequencing was able to recover more positive results than the traditional methods, it has several limitations, including the necessity for high bacterial loads (such that there is sufficient starting DNA to sequence) and the increased costs (in our study, we observed that at least 5 Gigabase pairs of sequencing data is required to get a basic amount of depth and sensitivity when looking for specific strains, roughly equivalent to 5x the required sequencing for a single isolate). In future, initial PCR from the environmental DNA targeting a known marker in the outbreak strain could help narrow the candidates for complete metagenomic sequencing. Further work is required to refine these methods and determine an accurate guideline, particularly as it relates to sequencing depth and sensitivity.

All of the positive sequencing and culture results from the environmental sampling were from areas presently or previously being used by patients colonized with the ST1050 CR-Ab. As such, we cannot be sure that the identified ST1050 CR-Ab was present in these environments prior to colonization, or if it was shed from the patient. Subsequent environmental sampling was carried out after each round of cleaning, and no CR-Ab was detected afterwards. It is most likely that the CR-Ab detected in the environmental reservoirs were shed from the patients, however this result does indicate the ease of transmission of this organism from colonized patients to fomites within the hospital, where they then might transmit to other areas or to hospital staff [49]. Burns baths are a particular risk to patients, as denuded skin is easily colonized and presents a high risk for subsequent infection. ‘Splash-back’ from sinks and/or drains where MDR bacteria, such as CR-Ab, reside could present a hypothetical route for reinfection and ongoing transmission.

## Conclusion

By using WGS to assist in a large outbreak of CR-Ab (and other MDR Gram-negative bacilli) we show how genomics can be used to improve rapid respond measures and outbreak management, as well as provide in-depth characterization of the outbreak strains to establish a historical database that can be used to guide responses to future outbreaks. We also show how direct sequencing of environmental samples was able to detect evidence of the outbreak strain leading to key changes in infection control policy.

## Supplementary Data

Supplementary material 1

Supplementary material 2

Supplementary material 3
