# Supplementary material for: Genomic surveillance, characterization and intervention of a polymicrobial multidrug-resistant outbreak in critical care
Source: Microb Genom. 2021 Feb 18;7(3):mgen000530. doi: 10.1099/mgen.0.000530 (PMC8190620; doi:10.1099/mgen.0.000530)
Supplement: Supplementary material 1 [file mgen-7-0530-s001.pdf]

# Supplementary Material

Supplementary Methods:

*Illumina sequencing:*

All isolates collected between May-September 2016 (and all historically collected isolates) were sequenced at the Australian Centre for Ecogenomics (ACE) Sequencing Service, University of Queensland, Brisbane, Australia. DNA was quantitated using Qubit and libraries prepared using Nextera XT library prep (Illumina) with Nextera XT/V2 Indexes, as per manufacturer's instructions. Resulting libraries were quantitated with either qPCR or Tapestation, pooled and each sample loaded onto either 1/100$^{th}$ or 1/200$^{th}$ of a flow cell and sequenced on the NextSeq (Illumina) using a 2 x 150bp High Output V2 kit.

All subsequent isolates (from October 2016 onwards) were sequenced at the Public Health Microbiology Laboratory at Queensland Forensic and Scientific Services, Brisbane, Australia. All libraries were prepared using the Nextera XT DNA preparation kit (Illumina) and sequencing was performed on a NextSeq 500 (Illumina) with 2x150bp chemistry, NextSeq Midoutput kit v2.5.

*Bioinformatic analysis for clinical reporting:*

Methodologies for bioinformatic analysis and communication of Illumina WGS data during primary outbreak in 2016 (June 22, July 16, Aug 2, Aug 29) are outlined below:

Quality control and *de novo* assembly of Illumina WGS data and comparative genome analysis were carried out as described in the main document. Raw reads were analysed using Nullarbor (https://github.com/tseemann/nullarbor) to determine MLST, antibiotic resistance gene profile, and core SNP phylogeny using species-specific reference sequences. Closest publicly available complete genomes were chosen as reference sequences where available (*Acinetobacter baumannii* Global Clone (GC) 2 strain 1656-2 (GI:384129960); *Klebsiella pneumoniae subsp. pneumoniae* MGH 78578 (GI:150953431); *Serratia marcescens* WW4 (GI:448239774); *Pseudomonas aeruginosa* PA1R (GI:558665962). For *Enterobacter cloacae* the reference was the concatenated draft genome of *Enterobacter cloacae* Ecl1 (GenBank: JRFQ01000000; now reassigned as *E. hormaechei*), an ST90 strain isolated from a burns patient at the RBWH ICU in 2015. Antibiotic resistance gene content and MLST was further checked using srst2[1] against the ARG-ANNOT[2] database and the Oxford MLST scheme[3], respectively. Plasmid Inc Typing was done based on the relaxase gene as described by Compain *et al.* [4].

SNP differences between strains were determined using Nesoni (https://github.com/Victorian-Bioinformatics-Consortium/nesoni) and evolutionary relationships were determined shown as phylograms or Eburst-style matrices in which nodes of identical isolates were separated by branches representing one or more core SNP differences radiating from a founder (index) node. This format provided consistency across reports, enabling a progressive expansion of the display figure from a common anchor as each WGS batch was reported, in contrast to phylograms where topology and isolate order could change substantially as the data was updated. For example Supplementary Figure 2 is a close approximation of the CR-Ab tree reported

in August 2016 and forms the anchor to Figure 3B which shows all ST1050 CR-Ab in the study.

Methodologies used for subsequent reports (Nov 4 2016, Mar 9 2017, Jun 20 2017, Oct 10 2017) were essentially the same except that the concatenated draft genome of ST1050 CR-Ab MS8436 or MS14413 was used as reference sequences, Abricate (v0.6) with ResFinder was used for antibiotic gene prediction, and from 2017 Nesoni implemented Bowtie for alignment instead of SHRiMP.

All 2018 CR-Ab isolate genomes were initially reported to RBWH as part of an Infectious Diseases demonstration project in WGS surveillance of MDR bacteria in hospitals (encompassing most of this authorship group) funded by the Queensland Genomics Health Alliance (now Queensland Genomics), Queensland State Government, Australia.

*Pacific Biosciences sequencing:*

One CR-Ab isolate (MS14413) and one *K. pneumoniae* isolate (MS14393) were selected for sequencing with Pacific Biosciences (PacBio) Single Molecule Real-Time (SMRT) sequencing. Isolates were grown on LB agar at 37°C overnight. A single colony was used to inoculate 10 ml LB broth, grown overnight at 37°C (shaking 250 rpm). DNA was extracted using the UltraClean® Microbial DNA Isolation Kit (MO BIO Laboratories) as per manufacturer's instructions. 20kb SMRTbell libraries were prepared using P6 polymerase and C4 sequencing chemistry with 7kb size selection with BluePippin. Final polymerase bound libraries were sequenced using 1 SMRT cell each on a PacBio RSII instrument at the University of Queensland Centre for Clinical Genomics, Translational Research Institute, Brisbane, Australia.

*Pacific Biosciences genome assembly and annotation:*

PacBio genomes were assembled using Canu[5] v1.3 and manually closed using Artemis[6]. A large duplicated region of ~40 kb was identified in the CR-Ab isolate and resolved using read-mapping and PCR at unique borders of the duplication (see below). The SMRT Analysis suite (v7.0.1.66975) was used to generate methylated motif summaries and polished assemblies using the PacBio reads. Indels were further corrected with Pilon[7] v1.22 using the trimmed Illumina reads mapped to the assembly using BWA[8] v0.7.16a-r1181. Complete PacBio genomes were annotated using Prokka[9] v1.12-beta. Insertion sequences were identified using ISSaga[10]. Frameshifts were corrected by manually inspecting read pileup at suspected positions identified using NCBI microbial genome submission check (https://www.ncbi.nlm.nih.gov/genomes/frameshifts/frameshifts.cgi).

*Closing the genome of CR-Ab strain MS14413:*

*De novo* assembly of the CR-Ab strain MS14413 PacBio reads using Canu resulted in two contigs: one large contig (~4 Mb) and one small contig (~65 kb). The large contig corresponded to the CR-Ab chromosome and could be circularized. Comparison of both contigs to each other using the Artemis Comparison Tool (ACT) determined that the smaller contig matched the ends of the chromosome perfectly except for a duplicated ~4.8 kb region. Rearrangement of this region onto the contig ends and re-mapping of both PacBio and Illumina reads to the chromosome (using BWA and blasr, respectively) resolved the ~4.8 kb region but simultaneously identified approximately twice as much read depth across a ~40 kb region on the chromosome, indicative of a large collapsed repeat in our initial assembly. Comparative analysis of the smaller

contig (using ACT) followed by PCR at unique borders of the suspected tandem duplication (Supplementary Figure 1, Supplementary Table 1) enabled resolution of the region as two copies of ~41 kb. Further mapping with both the Illumina and PacBio reads (using BWA[8] and blasr[11], respectively) confirmed the tandem duplication, which has been included in the complete genome of CR-Ab strain MS14413 (GenBank: CP054302.1).

**Supplementary Results:**

No transmission of *P. aeruginosa* or *S. marcescens* from the index patient was observed

*P. aeruginosa* isolates from the index patient were found to be ST979 and all carried 5 antibiotic resistance genes (*aph(3')-IIb*, *bla*OXA-50, *bla*PAO, *catB7* and *fosA*) (Supplementary Table 2). The final *P. aeruginosa* isolate from the index patient (MS14412) was found to be more resistant to carbapenems, likely due to a nonsense mutation in the outer membrane porin *oprD*, as well as a non-conservative amino acid change in the response regulator *parR*. Initial *S. marcescens* isolates appeared to only carry *aac(6')-Ic*, *bla*SRT-2, *oqxB*, and *qnrE*. However, later acquisition of an IncHI2 plasmid in two *S. marcescens* isolates was associated with large number of additional resistance genes, including the ESBL *bla*CTX-M-15 (Supplementary Table 3).

Genomic factors affecting adhesion, biofilm formation and motility in CR-Ab strains

Analysis of SNP differences between the ST1050 CR-Ab isolates found a single SNP resulting in a reversion of a nonsense mutation to a functional amino acid codon in the gene *filB* within the *fil* operon; a putative type 3 filamentous fimbriae. This reversion was obscured in the Snippy analysis, as one isolate (MS14422) was heterozygous at this position, resulting in it being masked from the core SNP analysis. This reversion corresponded to the latter half of the outbreak, including all CR-*Ab* isolates taken after the 4$^{th}$ of July 2016 (with the exception of MS14413 [6/7/16], MS14438 [12/9/16] and SS17M5036 [17/5/17]) (Supplementary Figure 4). As stated, MS14422 appeared to have both alleles at this position and represents either (i) a transitioning population reverting from a functional codon back to a stop codon, or (ii) a mixed population of

both allele types. Due to the unusual nature of this nonsense mutation reversion, we downloaded all available complete publicly available *A. baumannii* strains from NCBI (accessed 15-11-2018) and inspected the *filB* region. This analysis showed that there were multiple *A. baumannii* strains with disrupted *filB* genes, caused not only by nonsense mutations, but also interruption by insertion sequences and frameshift mutations (Supplementary Figure 4). Many of the publicly available strains also appeared to have functional *filB* genes, with reversion from a stop codon to a functional gene possible based on the phylogeny. While not much is known about this fimbriae in *A. baumannii*, several of the genes within this operon were shown to be down-regulated in community settings[12], suggesting that it may not be required in biofilms or stable bacterial populations. It is possible that it increases survivability or transmission throughout the environment, however further work is required to determine the phenotypic qualities of this mutation.

## Supplementary Tables:

**Supplementary Table 1:** Primers used to resolve large duplication in CR-Ab isolate MS14413

| Name | Sequence | Length | GC | Tm |
|---|---|---|---|---|
| 8076_A1_border1_F | TCCCGGCACTTAACTTACGT | 20 | 50 | 63.7 |
| 8077_A2_wbuB_R | CGGCCAATCACGATCAGATG | 20 | 55 | 69.2 |
| 8078_B1_OXA23_F | TCTGTATTTGCGCGGCTTAG | 20 | 50 | 65.4 |
| 8079_C1_border2_R | TTAGCTCCCCACACACTGAG | 20 | 55 | 63.4 |

**Supplementary Table 2:** Antibiotic resistance genes in *P. aeruginosa* isolates from the index patient: tick denotes presence

| | aph(3')-IIb_1 | blaOXA-50_1 | blaPAO_4 | catB7_1 | fosA_1 |
|---|---|---|---|---|---|
| MS14395 | ✓ | ✓ | ✓ | ✓ | ✓ |
| MS14399 | ✓ | ✓ | ✓ | ✓ | ✓ |
| MS14403 | ✓ | ✓ | ✓ | ✓ | ✓ |
| MS14412 | ✓ | ✓ | ✓ | ✓ | ✓ |

**Supplementary Table 3:** Antibiotic resistance genes in *S. marcescens* isolates from the index patient: tick denotes presence; number denotes %nucleotide coverage (where two numbers separated by a comma represents a split blast result)

| | **QnrB1_1** | **aac(3)-IIa_1** | **aac(6')-Ic_1** | **aac(6')Ib-cr_1** | **aadA1_1** | **blaCTX-M-15_23** | **blaOXA-1_1** | **blaSRT-2_1** | **blaTEM-1B_1** | **catA1_1** | **dfrA14_1** | **oqxB_1** | **qnrE_1** | **strA_4** | **strB_1** | **sul2** | **tet(A)_4** |
|---|---|---|---|---|---|---|---|---|---|---|---|---|---|---|---|---|---|
| **MS8415** | | | ✓ | | | | | ✓ | | | | ✓ | ✓ | | | | |
| **MS14404*** | ✓ | | ✓ | ✓ | ✓ | ✓ | 51.74, 19.01 | ✓ | ✓ | 65.91 | ✓ | ✓ | ✓ | ✓ | ✓ | ✓ | ✓ |
| **MS8416*** | ✓ | ✓ | ✓ | ✓ | ✓ | ✓ | 40.07, 52.23 | ✓ | ✓ | ✓ | ✓ | ✓ | ✓ | ✓ | ✓ | ✓ | ✓ |

* Contain IncHI2 plasmid

**Supplementary table 4: Positive culture and sequencing results from 2018 environmental sampling**

| **Sample** | **Isolation date** | **Culture result** | **Sequencing result** | **# reads total in sample** | **# reads mapped to MS14413** | **% reads** | **# Contigs in *de novo* assembly** |
|---|---|---|---|---|---|---|---|
| R5666 | 16/08/2018 | ST1050 CR-Ab | No CR-Ab | n/a | n/a | n/a | n/a |
| R5515 | 26/07/2018 | No CR-Ab | ST1050 CR-Ab | 43582216 | 444810 | 1.0% | 4173 |
| R5510 | 26/07/2018 | No CR-Ab | ST1050 CR-Ab | 51421258 | 3134890 | 6.1% | 8125 |
| R5863 | 02/10/2018 | No CR-Ab | ST1050 CR-Ab | 41402234 | 369031 | 0.9% | 10011 |
| R5864 | 02/10/2018 | ST1050 CR-Ab | ST1050 CR-Ab | 38635392 | 2634975 | 6.8% | 1326 |

## Supplementary Figures:

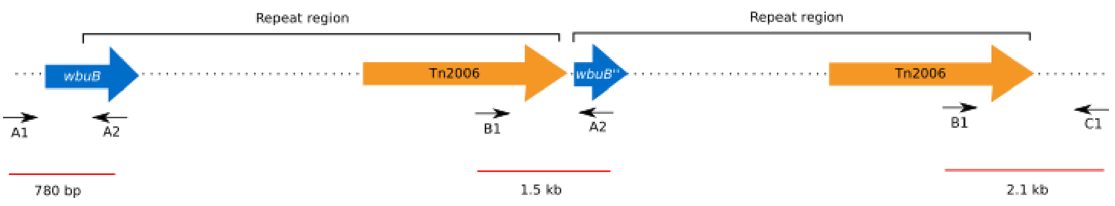


**Supplementary Figure 1:** Primer binding regions to resolve large duplication in CR-Ab isolate MS14413 (not to scale)

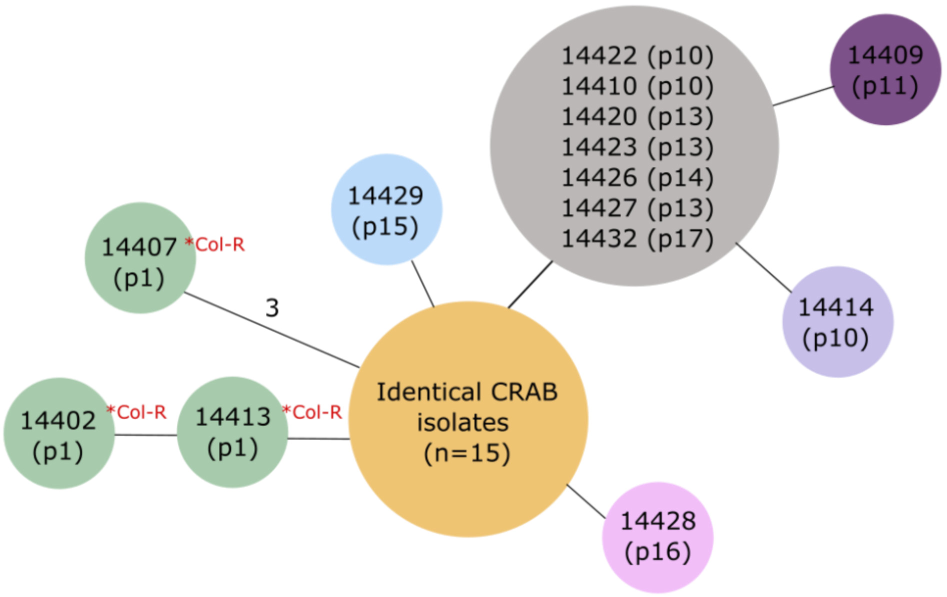


**Supplementary Figure 2: Relationship matrix of outbreak ST1050 CR-Ab isolates from 2016:** isolates within the same circle are identical at the core SNP level. Black lines indicate 1 SNP difference, except where stated otherwise. Patient numbers denoted with "p" and the corresponding number. "Col-R" denotes isolates with colistin resistant SNPs. The yellow circle includes 15 isolates identical at the core SNP level from patients 1-9 and 12: MS14431, MS14430, MS14397 (p1); MS8410, MS8412 (p2); MS8422 (p3); MS8413 (p4); MS8419 (p5); MS8436, MS8440 (p6); MS8442 (p7) MS8441 (p8), MS14388 (p9) and MS14421 (p12).

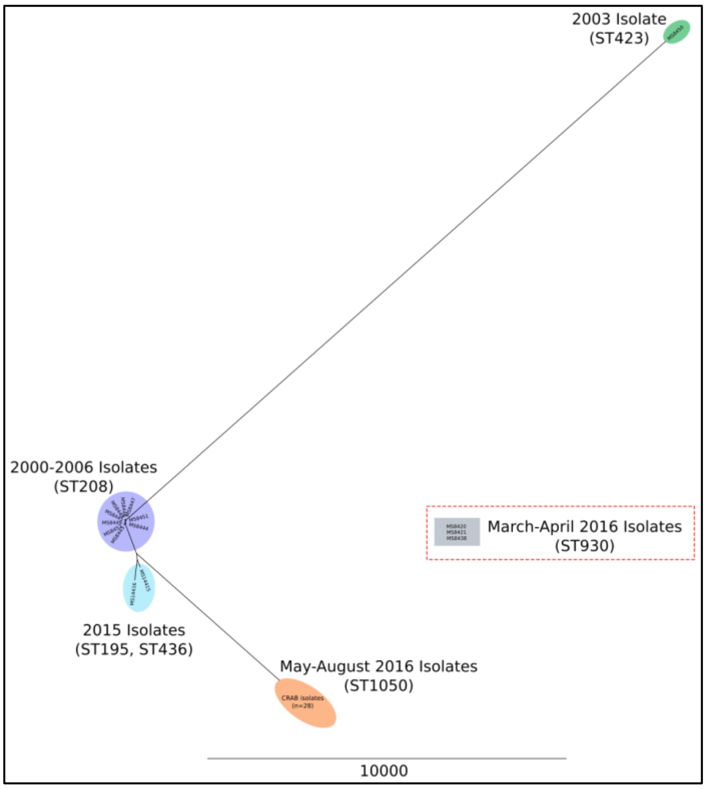


**Supplementary Figure 3: Comparison of outbreak ST1050 CR-Ab isolates to CR-Ab previously isolated from the same hospital between 2000 to early 2016:** Tree was created with Parsnp v1.2 (default settings) using the draft *de novo* assemblies for the ST1050 CR-Ab as well as 17 historical CR-Ab from the same hospital. For clarity, the node representing the March-April 2016 isolates (ST930) has been removed from tree as its long branch length obscured the other nodes (see red box inset).

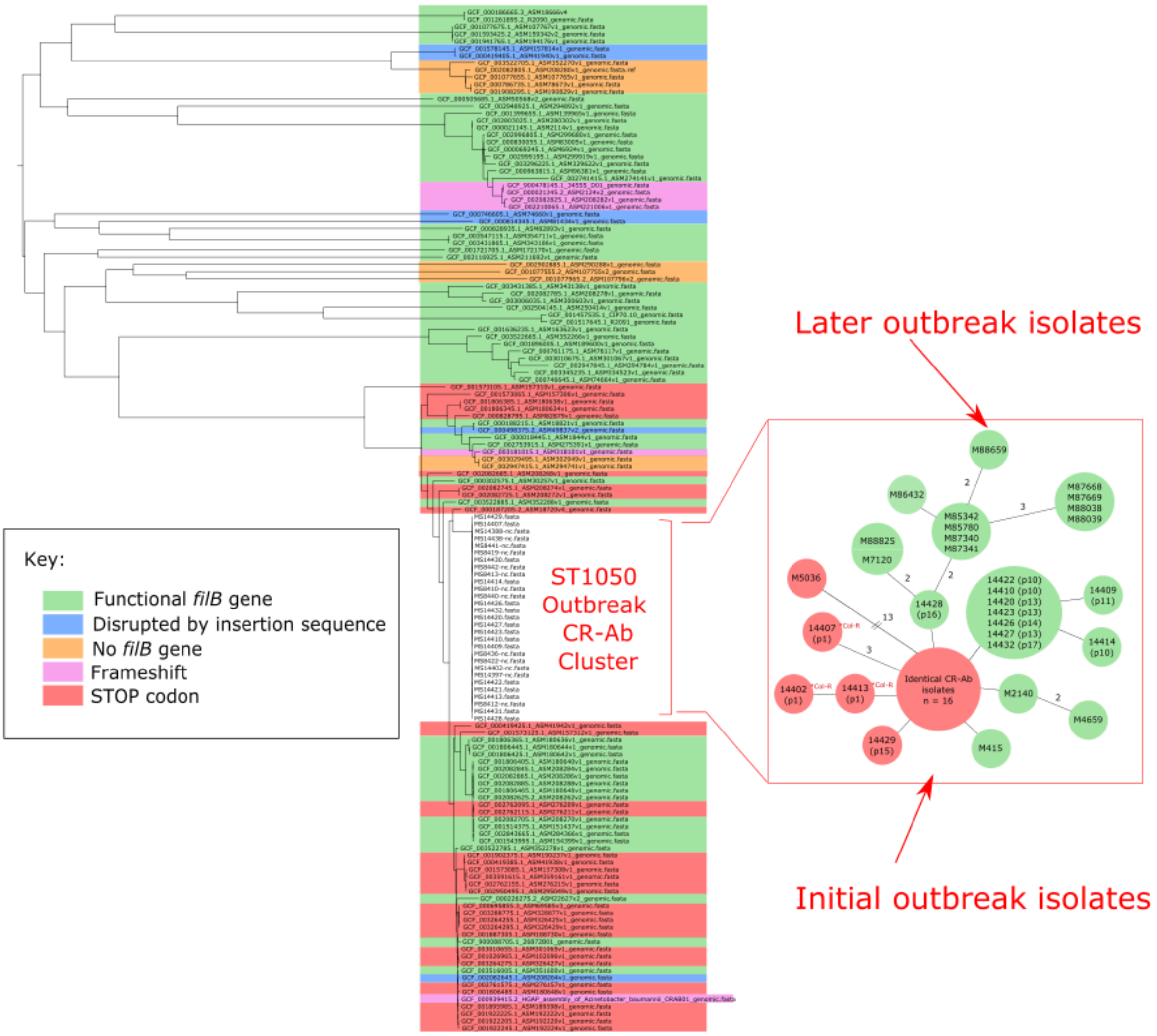


**Supplementary Figure 4: Analysis of nonsense mutation reversion in ST1050 outbreak CR-Ab and complete publicly available *A. baumannii* (from NCBI):** Tree built using Parsnp v1.2 (under default settings) with 113 complete *A. baumannii* and the initial outbreak ST1050 CR-Ab genomes (mid-point rooted). Taxa are coloured according to *filB* genotype (refer to key). Inset box shows relationship matrix from Figure 3 (in main text) with nodes coloured according to *filB* genotype. SS17M414 (isolated 3/1/2017) also has a functional *filB* gene, however, is not displayed in the relationship matrix as it clusters in the large group of 16 identical isolates.

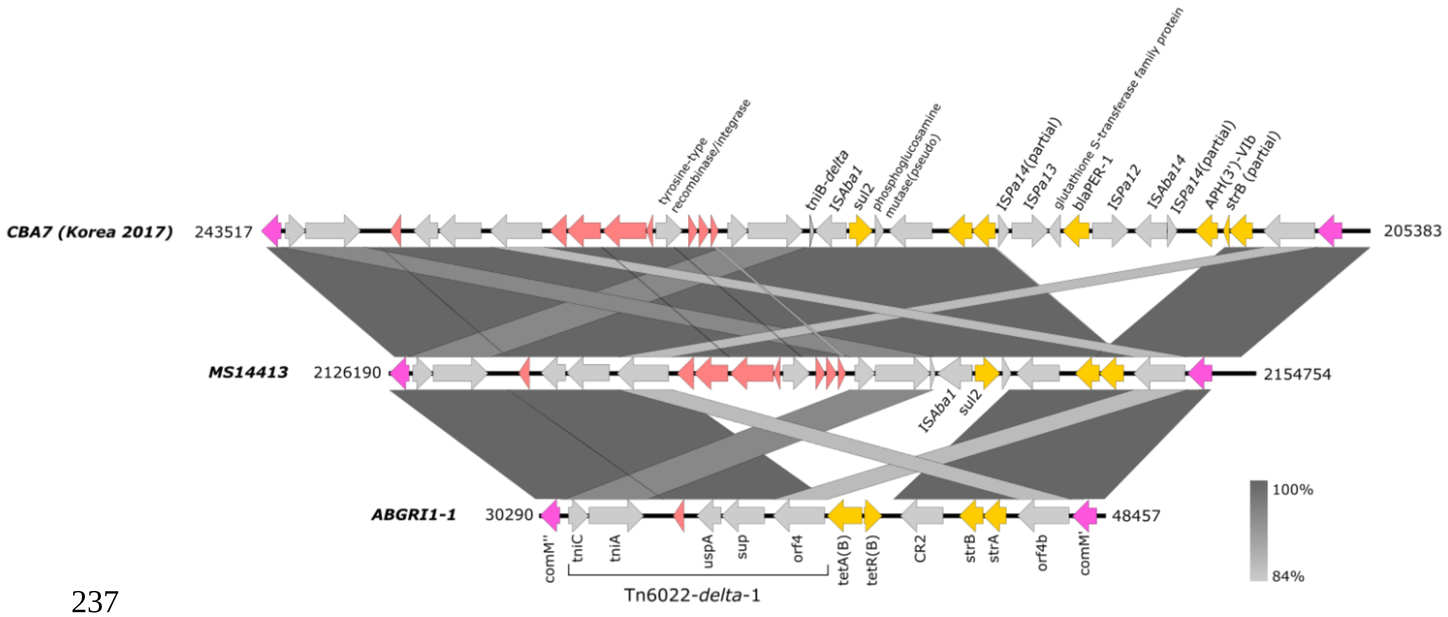


**Supplementary Figure 5: Novel AbGRI1 resistance island in MS14413.** Top: *A. baumannii* strain CBA7 (CP020586.1), isolated in Korea 2017. Middle: *A. baumannii* ST1050 outbreak strain MS14413 (CP054302.1). Bottom: Antibiotic resistance island ABGRI1-1 (Tn6166; JN247441.1). ComM gene (disrupted) coloured pink. Resistance genes coloured yellow. Hypothetical genes coloured red. All other genes coloured grey, with associated feature labelled. Figure generated using Easyfig. BLASTn comparison shown as grey bars.

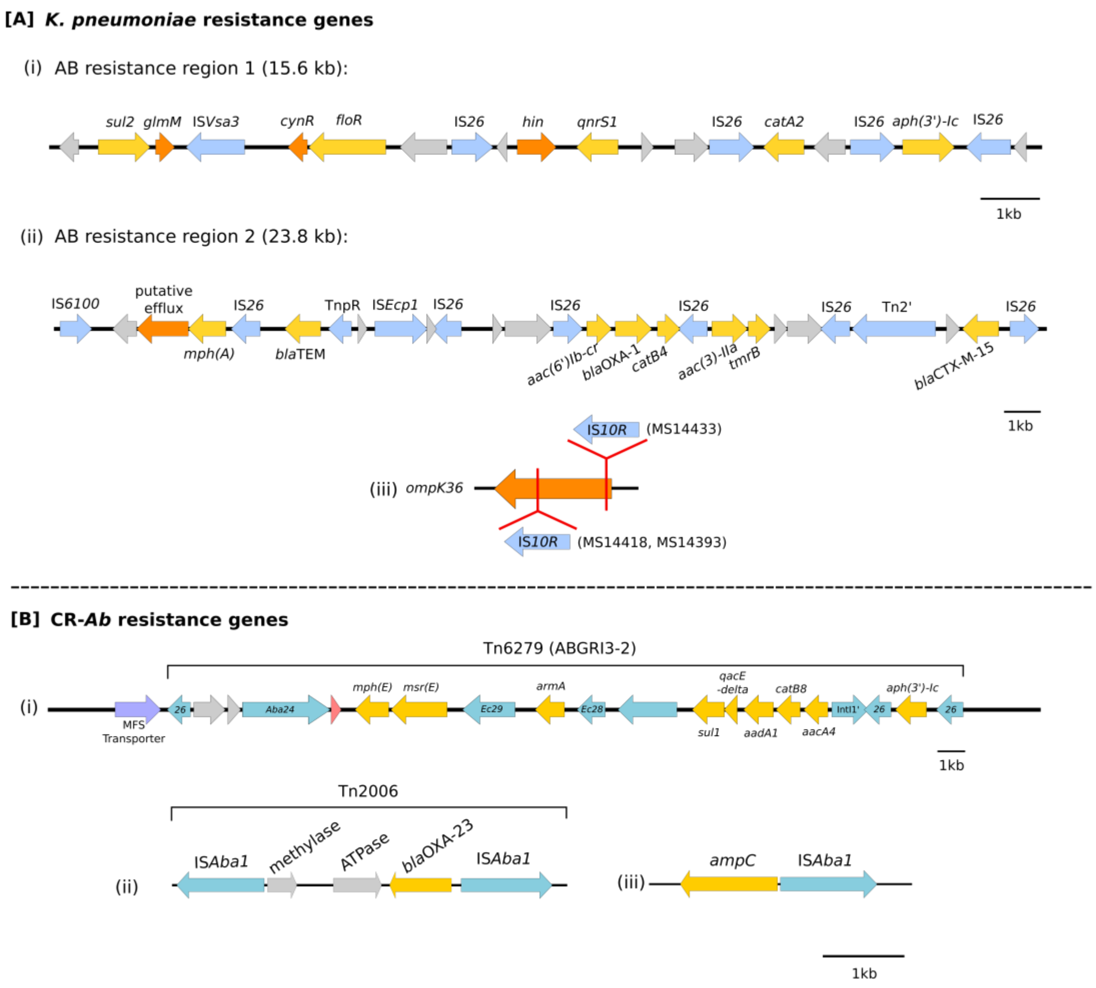


**Supplementary Figure 6: Resistance regions found in *K. pneumoniae* [A] and CR-*Ab* [B] isolates:** arrows represent CDS. Colours represent resistance genes (yellow), regulatory/efflux genes (orange), mobile elements (blue) and hypothetical genes (grey). [A] two resistance regions were found in all *K. pneumoniae* isolates, located on an IncA/C plasmid. [B] CR-*Ab* isolates were found to have three main mechanisms of resistance: (i) a large transposon Tn6279 (ABGRI3-2), (ii) a smaller transposon (Tn2006) and (iii) an IS*Aba1* element upstream of the intrinsic *ampC* gene.

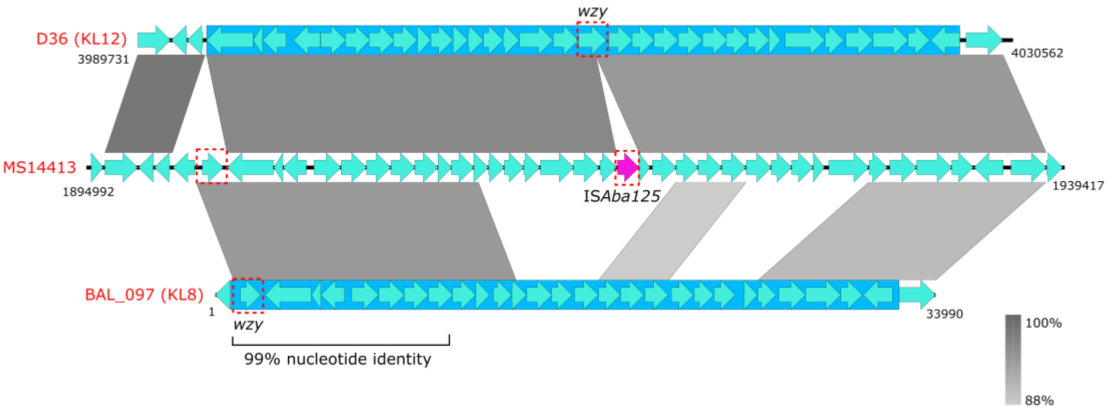


**Supplementary Figure 7: *wzy* gene positions in capsule (K) region:** Light blue arrows represent CDS regions. Dark blue box represents capsule region. Outbreak CR-*Ab* isolate MS14413 was found to have a 97% nucleotide similarity to the KL12 capsule (K) locus found in the GC1 *A. baumannii* strain D36 (GenBank:NZ_CP012952.1) except for an IS*Aba125* insertion sequence in the *wzy* gene. Further comparison found a second *wzy* gene in the same position as in the *A. baumannii* strain BAL_097 (GenBank: KX712116).

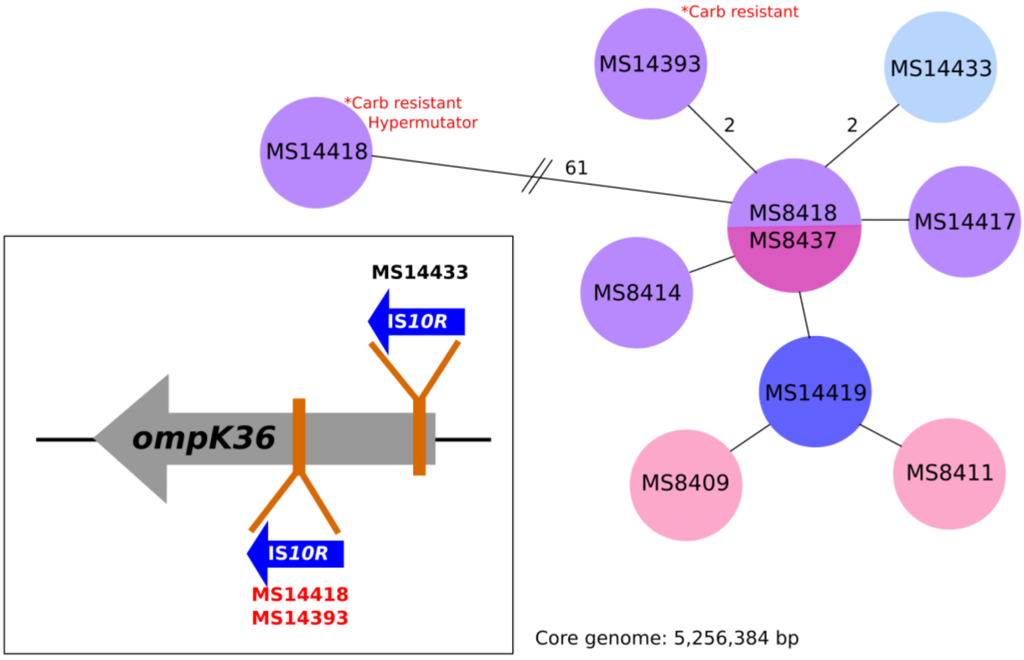


**Supplementary Figure 8: Relationship matrix of ST515 *Klebsiella pneumoniae* isolates during 2016 outbreak and disruption of *ompK36* outer membrane porin by IS*10R*:** coloured circles correspond to patient. Branches represent one SNP difference unless otherwise stated.

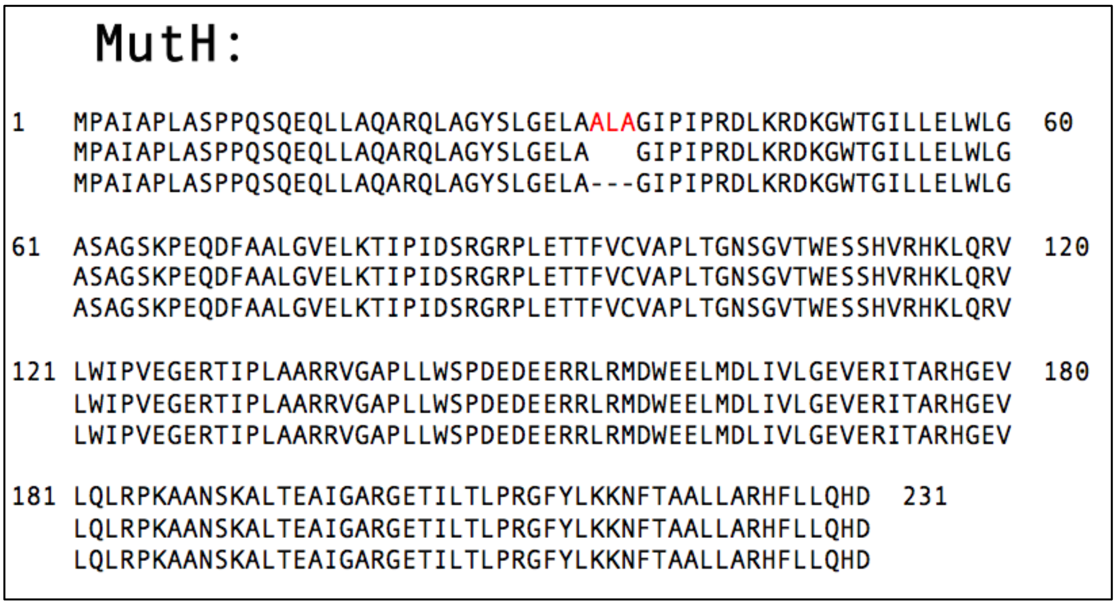


**Supplementary Figure 9: Deletion of three amino acids from MutH protein in MS14418**

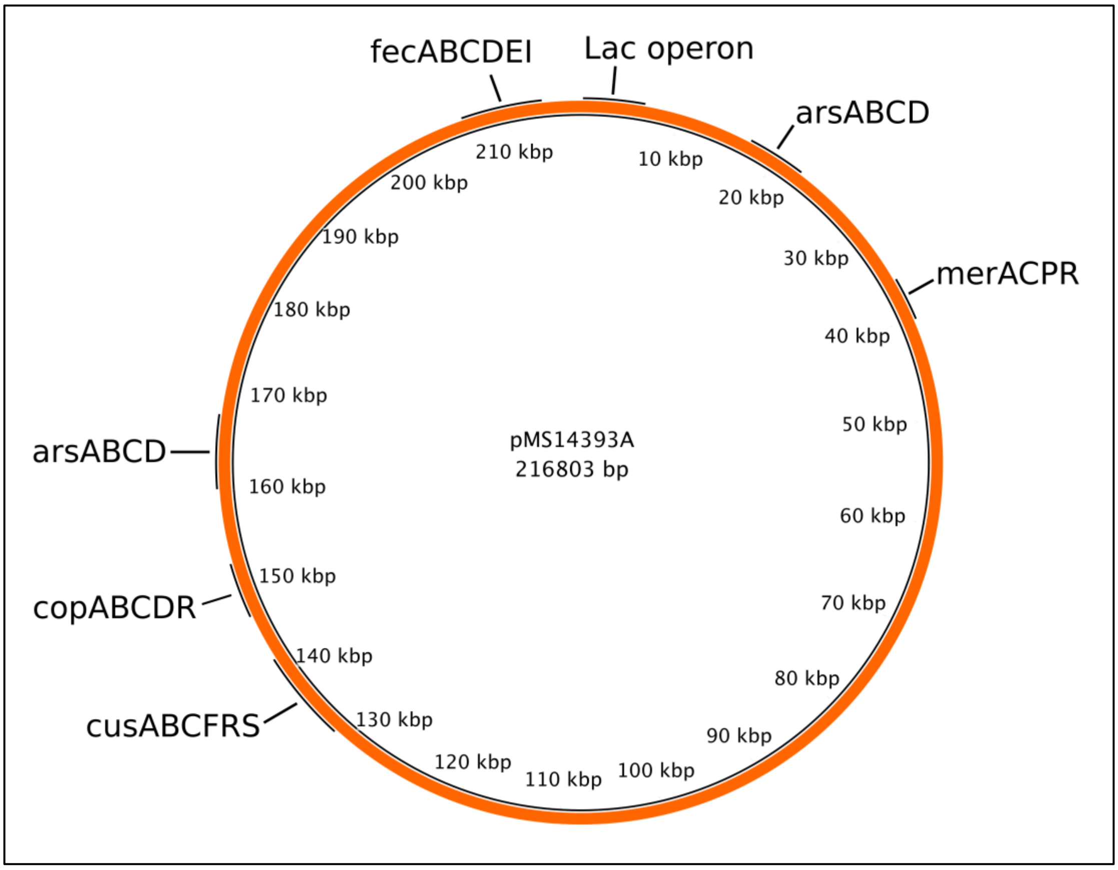


**Supplementary figure 10: PacBio assembly of IncF plasmid carried by *K. pneumoniae* isolate MS14393**

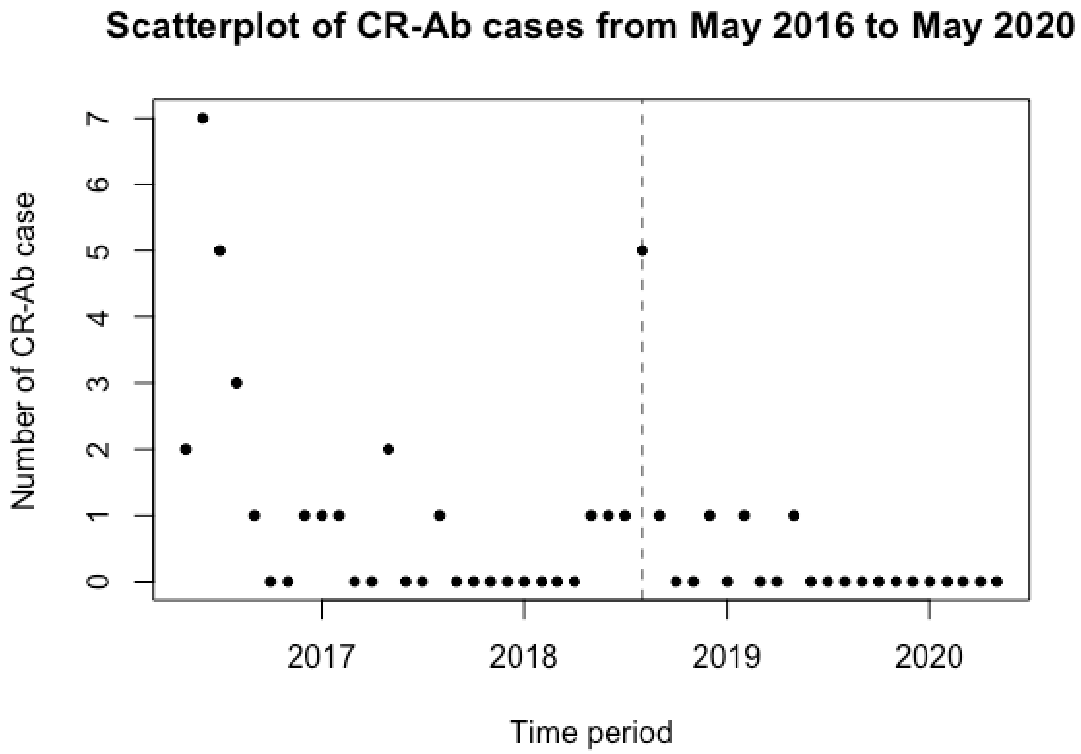


**Supplementary Figure 11: Incidence of ST1050 CR-Ab cases from the time of the initial outbreak until May 2020.**

References:

1. Inouye M, Dashnow H, Raven LA, Schultz MB, Pope BJ, Tomita T, Zobel J, Holt KE: **SRST2: Rapid genomic surveillance for public health and hospital microbiology labs**. *Genome Med* 2014, **6**(11):90.
2. Gupta SK, Padmanabhan BR, Diene SM, Lopez-Rojas R, Kempf M, Landraud L, Rolain JM: **ARG-ANNOT, a new bioinformatic tool to discover antibiotic resistance genes in bacterial genomes**. *Antimicrob Agents Chemother* 2014, **58**(1):212-220.
3. Bartual SG, Seifert H, Hippler C, Luzon MA, Wisplinghoff H, Rodriguez-Valera F: **Development of a multilocus sequence typing scheme for characterization of clinical isolates of Acinetobacter baumannii**. *J Clin Microbiol* 2005, **43**(9):4382-4390.
4. Compain F, Poisson A, Le Hello S, Branger C, Weill FX, Arlet G, Decre D: **Targeting relaxase genes for classification of the predominant plasmids in Enterobacteriaceae**. *Int J Med Microbiol* 2014, **304**(3-4):236-242.
5. Koren S, Walenz BP, Berlin K, Miller JR, Bergman NH, Phillippy AM: **Canu: scalable and accurate long-read assembly via adaptive k-mer weighting and repeat separation**. *Genome Res* 2017, **27**(5):722-736.
6. Carver T, Harris SR, Berriman M, Parkhill J, McQuillan JA: **Artemis: an integrated platform for visualization and analysis of high-throughput sequence-based experimental data**. *Bioinformatics* 2012, **28**(4):464-469.
7. Walker BJ, Abeel T, Shea T, Priest M, Abouelliel A, Sakthikumar S, Cuomo CA, Zeng Q, Wortman J, Young SK *et al*: **Pilon: an integrated tool for comprehensive microbial variant detection and genome assembly improvement**. *PLoS One* 2014, **9**(11):e112963.
8. Li H, Durbin R: **Fast and accurate short read alignment with Burrows-Wheeler transform**. *Bioinformatics* 2009, **25**(14):1754-1760.
9. Seemann T: **Prokka: rapid prokaryotic genome annotation**. *Bioinformatics* 2014, **30**(14):2068-2069.
10. Varani AM, Siguier P, Gourbeyre E, Charneau V, Chandler M: **ISsaga is an ensemble of web-based methods for high throughput identification and semi-automatic annotation of insertion sequences in prokaryotic genomes**. *Genome Biol* 2011, **12**(3):R30.
11. Chaisson MJ, Tesler G: **Mapping single molecule sequencing reads using basic local alignment with successive refinement (BLASR): application and theory**. *BMC Bioinformatics* 2012, **13**:238.
12. Miller DP, Wang Q, Weinberg A, Lamont RJ: **Transcriptome analysis of Porphyromonas gingivalis and Acinetobacter baumannii in polymicrobial communities**. *Mol Oral Microbiol* 2018, **33**(5):364-377.

**School of Chemistry and Molecular Biosciences**

A/Prof Scott Beatson
Head of Microbial Genomics Group
Australian Infectious Diseases Research Centre
Australian Centre for Ecogenomics

June 22, 2016

**<u>Preliminary report</u>: Genome sequencing of Carbapenem-resistant *Acinetobacter baumannii* (CRAB) and companion isolates from RBWH (version 1)**

***Summary:*** Genome sequencing of 18 clinical isolates (11 carbapenem-resistant *Acinetobacter baumannii*, 6 *K. pneumoniae* and 1 carbapenem-resistant *Pseudomonas aeruginosa*) was carried out at The University of Queensland (UQ). These were cultured from patients at RBWH, 6 involved in the current suspected CRAB outbreak and 3 identified earlier in 2016 as CRAB-colonised or infected. Two historical CRAB strains from 2006 were also sequenced for comparison.

**Two separate introductions of CRAB into the ICU / Burns Unit have occurred in 2016**. The first in **March-April** involved 3 confirmed cases infected or colonized with **closely related ST930 strains carrying an OXA-134 carbapenemase**. The more recent introduction in **May-June** involved a divergent **ST1050** strain belonging to the **International Clone 2 (IC2), and carried OXA-23 carbapenemase**. This has been confirmed by genomics in 5 patients from the current outbreak during May-June 2016, although a further 3 cases remain to be analysed. These current strains tightly cluster by single nucleotide polymorphism (SNP) profiling, confirming transmission between patients. To date, no CRAB strains have yet been identified from the environmental sampling. **All 2016 clusters were different from the 2006 outbreak strains, which were ST208 with OXA-23**.

Simultaneously there has been evidence of **transmission between 4 patients in the current outbreak of an ESBL (CTX-M-15)-producing *Klebsiella pneumoniae*** which are also closely related (all are ST515). There was no evidence of inter-species transmission of carbapenemase genes. **The single carbapenem-resistant *Pseudomonas aeruginosa* isolated in blood from one patient did not carry any acquired carbapenemase genes of significance**.

This preliminary report will be updated with further analysis and results following additional sequencing of strains delivered to UQ after preparation of the first batch of samples.

**This report is for research use only** – methods described here are not NATA accredited.

**Table 1:** Samples: The following were delivered and sequenced at UQ on the Illumina NextSeq instrument, commencing on the evening of 20th June

| Strain | Lab no | MRN | Initials | Species | Site | Date | Ward |
|---|---|---|---|---|---|---|---|
| **MS8409** | [redacted] | [redacted] | [redacted] | K. pneumoniae (ESBL) | Urine | 4/06/2016 | [redacted] |
| **MS8410** | [redacted] | [redacted] | [redacted] | CR-A. baumannii | Blood | 4/06/2016 | [redacted] |
| **MS8411** | [redacted] | [redacted] | [redacted] | K. pneumoniae (ESBL) | Rectal swab | 30/05/2016 | [redacted] |
| **MS8412** | [redacted] | [redacted] | [redacted] | CR-A. baumannii | Rectal swab | 30/05/2016 | [redacted] |
| **MS8413** | [redacted] | [redacted] | [redacted] | CR-A. baumannii | Wound swab | 6/06/2016 | [redacted] |
| **MS8414** | [redacted] | [redacted] | [redacted] | CR-A. baumannii * | Rectal swab | 30/05/2016 | [redacted] |
| **MS8417** | [redacted] | [redacted] | [redacted] | K. pneumoniae (ESBL) | Blood | 7/05/2016 | [redacted] |
| **MS8418** | [redacted] | [redacted] | [redacted] | K. pneumoniae (ESBL) | Blood | 6/05/2016 | [redacted] |
| **MS8419** | [redacted] | [redacted] | [redacted] | CR-A. baumannii | ETA | 6/06/2016 | [redacted] |
| **MS8420** | [redacted] | [redacted] | [redacted] | CR-A. baumannii | Wound swab | 20/04/2016 | [redacted] |
| **MS8421** | [redacted] | [redacted] | [redacted] | CR-A. baumannii | Blood | 20/03/2016 | [redacted] |
| **MS8422** | [redacted] | [redacted] | [redacted] | CR-A. baumannii | ETA | 5/06/2016 | [redacted] |
| **MS8423** | [redacted] | [redacted] | [redacted] | K. pneumoniae (non-ESBL) | ETA | 6/06/2016 | [redacted] |
| **MS8436** | [redacted] | [redacted] | [redacted] | CR-A. baumannii | Tissue (buttock) | 10/06/2016 | [redacted] |
| **MS8437** | [redacted] | [redacted] | [redacted] | K. pneumoniae (ESBL) | Urine | 8/06/2016 | [redacted] |
| **MS8438** | [redacted] | [redacted] | [redacted] | CR-A. baumannii | Blood | 6/03/2016 | [redacted] |
| **MS8439** | [redacted] | [redacted] | [redacted] | Psedomonas aeruginosa (mero-R) | Blood | 9/06/2016 | [redacted] |
| **MS8440** | [redacted] | [redacted] | [redacted] | CR-A. baumannii | Blood | 12/06/2016 | [redacted] |

*Note: identification incorrect, confirmed as *K. pneumoniae* by WGS – original isolate to be retrieved and re-sequenced

## GENOME ANALYSIS RESULTS:

**Carbapenem-resistant *A. baumannii* (CRAB)**

All the *A. baumannii* **strains from the current ICU cluster (from May to June 2016) are closely related to each other**, and belong to the **International Clone 2 (IC2)**, which is commonly seen in Asia and Oceania [1], and are **sequence type (ST) 1050** according to the Oxford MLST scheme. This is in contrast to a smaller cluster of **3 cases for March-April 2016, separated by almost 50,000 SNPs** to the more recent outbreak strains (see SNP tree image in Figure 1) and type as **ST930**. **This confirms two separate introductions of CRAB into the hospital**. It is noted that the **March-April 2016 cluster are closely related** to each other, suggesting limited transmission at the time. It is also notable that **both clusters are different from the strains cultured in the 2006 outbreak**, which belong to **ST208.**

The strains all carry between 13-14 acquired resistance genes (see Table 2). The **May-June cluster strains and the 2006 strains carried the carbapenemase OXA-23** (a carbapenemase gene commonly seen in CR-A. *baumannii*[2,3]), whereas these were absent in the March-April cluster. The **March-April strains carried the OXA-134 carbapenemase**, which is usually acquired from *Acinetobacter lwoffii*,[4] as well as an **IMP-26 carbapenemase** – this is of significance, as the IMP gene appears closely related to the IMP-4-like carbapenemase commonly seen in Queensland *Enterobacter* strains.[5] This will undergo additional genomic investigation. All *A. baumannii* possess various OXA-51-like beta-lactamases, which may confer carbapenem resistance if highly expressed.[2] All *A. baumanii* carry an *ampC* beta-lactamase, ADC-25, which may be expressed at high level by the addition of an insertion sequence (IS*Aba*1) in proximity to the gene, conferring resistance to cephalosporins (and will be sought during ongoing analysis of the data).[6]

It is also notable that recent strains carried the ***armA* methylase gene**, which confers broad aminoglycoside resistance (including amikacin), which conforms to the high MICs observed for aminoglycosides in many of these strains.

All strains also carried additional **resistance genes for antibiotic classes such as sulphonamides, chloramphenicol and macrolides/lincosamides**. The March-April 2016 strains also possessed resistance genes for **folate inhibitors (such as trimethoprim) and rifampicin**. In contrast to the 2006, recent 2016 isolates have no resistance genes to tetracyclines (also reflected in the MIC data).

Fluoroquinolone resistance mutations are not readily detected by most automated resistance gene annotation pipelines, and will require some additional data analysis to be confirmed.

**Table 2: Sequence type and antibiotic resistance gene profiles of CR-*A. baumannii***

| Strain | Case | ST | Species | Aminoglycoside | | | | | | | Beta-lactamase | | | | | | Phenicols | | Folate | $MLS_B$ | | Sulphonamide | | Tetracycline | Rifampicin |
|---|---|---|---|---|---|---|---|---|---|---|---|---|---|---|---|---|---|---|---|---|---|---|---|---|---|
| | | | | aac(3)-I | aac(3)-Ia | aadA1 | aph(3')-Ic | armA | strA | strB | blaADC-25 | blaIMP-26 | blaOXA-134 | blaOXA-23 | blaOXA-51-like | blaTEM-1D | catB8 | floR | dfrA18 | mph(E) | msr(E) | sul1 | sul2 | tet(B) | ARR-3 |
| MS1968 | - | 208 | *A. baumannii* | ✔ | ✔ | ✓ | ✔ | . | ✔ | ✔ | ✔ | . | . | ✔ | ✔ | ✔ | . | . | . | . | . | ✔ | ✔ | ✔ | . |
| MS1984 | - | 208 | *A. baumannii* | ✔ | ✔ | ✓ | ✔ | . | ✔ | ✔ | ✔ | . | . | ✔ | ✔ | ✔ | . | . | . | . | . | ✔ | ✔ | ✔ | . |
| MS8410 | [redacted] | 1050 | *A. baumannii* | . | . | ✓ | ✔ | ✓ | ✔ | ✔ | ✔ | . | . | ✔ | ✔ | . | ✔ | . | . | ✔ | ✔ | ✔ | ✔ | . | . |
| MS8412 | [redacted] | 1050 | *A. baumannii* | . | . | ✓ | ✔ | ✓ | ✔ | ✔ | ✔ | . | . | ✔ | ✔ | . | ✔ | . | . | ✔ | ✔ | ✔ | ✔ | . | . |
| MS8413 | [redacted] | 1050 | *A. baumannii* | . | . | ✓ | ✔ | ✓ | ✔ | ✔ | ✔ | . | . | ✔ | ✔ | . | ✔ | . | . | ✔ | ✔ | ✔ | ✔ | . | . |
| MS8419 | [redacted] | 1050 | *A. baumannii* | . | . | ✓ | ✔ | ✓ | ✔ | ✔ | ✔ | . | . | ✔ | ✔ | . | ✔ | . | . | ✔ | ✔ | ✔ | ✔ | . | . |
| MS8420 | [redacted] | 930 | *A. baumannii* | . | . | . | . | . | ✔ | ✔ | ✔ | ✔ | ✔ | . | ✔ | . | . | ✔ | ✔ | ✔ | ✔ | ✔ | ✔ | . | ✔ |
| MS8421 | [redacted] | 930 | *A. baumannii* | . | . | . | . | . | ✔ | ✔ | ✔ | ✔ | ✔ | . | ✔ | . | . | ✔ | ✔ | ✔ | ✔ | ✔ | ✔ | . | ✔ |
| MS8422 | [redacted] | 1050 | *A. baumannii* | . | . | ✓ | ✔ | ✔ | ✔ | ✔ | ✔ | . | . | ✔ | ✔ | . | ✔ | . | . | ✔ | ✔ | ✔ | ✔ | . | . |
| MS8436 | [redacted] | 1050 | *A. baumannii* | . | . | ✓ | ✔ | ✔ | ✔ | ✔ | ✔ | . | . | ✔ | ✔ | . | ✔ | . | . | ✔ | ✔ | ✔ | ✔ | . | . |
| MS8438 | [redacted] | 930 | *A. baumannii* | . | . | . | . | . | ✔ | ✔ | ✔ | ✔ | ✔ | . | ✔ | . | . | ✔ | ✔ | ✔ | ✔ | ✔ | ✔ | . | ✔ |
| MS8440 | [redacted] | 1050 | *A. baumannii* | . | . | ✓ | ✔ | ✔ | ✔ | ✔ | ✔ | . | . | ✔ | ✔ | . | ✔ | . | . | ✔ | ✔ | ✔ | ✔ | . | . |

**2006 Cluster**
**March-April 2016 Cluster**
**May-June 2016 Cluster**

✔ **Presence confirmed**
✓ **Requires confirmation**
. **Absent**

**This report is for research use only** – methods described here are not NATA accredited.

**Figure 1: SNP tree for *A. baumannii* strains isolated from patients in May-June 2016, March-April 2016 and 2006 clusters**

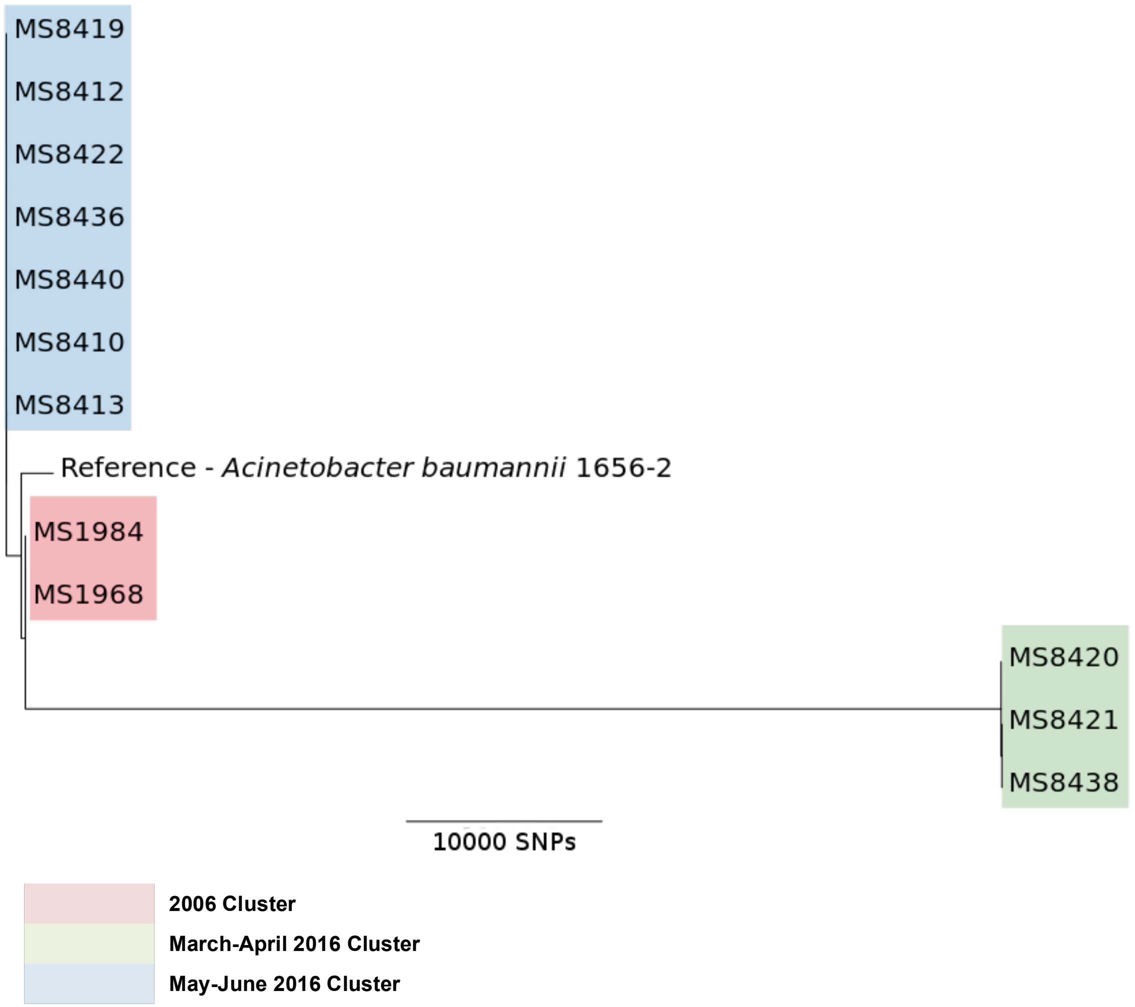


**This report is for research use only** – methods described here are not NATA accredited.

**_K. pneumoniae_ (ESBL-producer)**

All the *K. pneumoniae* strains identified as companion isolates from CRAB infected or colonised patients **were also found to be closely related**, being all of the same sequence type **(ST515)** and were similar by SNP profiling (details to follow). All carried 15 identical resistance genes, including the common **CTX-M-15 type ESBL**, as well as OXA-1, TEM-1 and SHV-1 (as always seen in *K. pneumoniae*) narrow spectrum beta-lactamases. However, if these latter genes are highly expressed, they may further contribute to broad-spectrum beta-lactam resistance.

**This confirms that transmission of this significant pathogen between patients was likely to have occurred in parallel to the CRAB outbreak**.

**Table 3: Sequence type and antibiotic resistance gene profiles of *K. pneumoniae***

| | | | | Aminoglycoside | | | Beta-lactamase | | | | Phenicols | | | Fosfomycin | $MLS_B$ | Quinolones | | Sulphonamides |
|---|---|---|---|---|---|---|---|---|---|---|---|---|---|---|---|---|---|---|
| Strain | Case | Species | ST | aac(3)-IIa | aac(6')Ib-cr | aph(3')-Ic | blaCTX-M-15 | blaOXA-1 | blaSHV-1 | blaTEM-1B | catA2 | catB3 | floR | fosA | mph(A) | oqxA | QnrS1 | sul2 |
| MS8409 | [redacted] | *K. pneumoniae* | 515 | ✔ | ✔ | ✔ | ✔ | ✔ | ✔ | ✔ | ✔ | ✔ | ✔ | ✔ | ✔ | ✔ | ✔ | ✔ |
| MS8411 | [redacted] | *K. pneumoniae* | 515 | ✔ | ✔ | ✔ | ✔ | ✔ | ✔ | ✔ | ✔ | ✔ | ✔ | ✔ | ✔ | ✔ | ✔ | ✔ |
| MS8418 | [redacted] | *K. pneumoniae* | 515 | ✔ | ✔ | ✔ | ✔ | ✔ | ✔ | ✔ | ✔ | ✔ | ✔ | ✔ | ✔ | ✔ | ✔ | ✔ |
| MS8423 | [redacted] | *K. pneumoniae* | 515 | ✔ | ✔ | ✔ | ✔ | ✔ | ✔ | ✔ | ✔ | ✔ | ✔ | ✔ | ✔ | ✔ | ✔ | ✔ |
| MS8437 | [redacted] | *K. pneumoniae* | 515 | ✔ | ✔ | ✔ | ✔ | ✔ | ✔ | ✔ | ✔ | ✔ | ✔ | ✔ | ✔ | ✔ | ✔ | ✔ |

**Carbapenem-resistant *Pseudomonas aeruginosa***

This single isolate was shown to contain an OXA-50 type beta-lactamase, which is a naturally occurring enzyme found in *P. aeruginosa* and is usually of limited clinical significance[7]. In addition to *aph(3')-IIb* aminoglycoside and *catB7* chloramphenicol resistance genes were present. **There was no evidence of carbapenemase acquired from the *A. baumannii* strains.** It is most likely that the multi-drug resistant (including to carbapenems) phenotype results from multiple chromosomal mutations, which will be investigated further.

**METHODS:**

***Bacterial culture**:* Isolates were delivered to the Schembri Lab at UQ in 2 stages (10th and 15th June 2016). Plate cultures were completely resuspended in 2 ml luria-bertani (LB) broth. 500 µl of this resuspension was used to inoculate 5ml LB broth, which was incubated for 1 hour at 37 degrees Celsius. Genomic DNA was then extracted using MoBio UltraClean® Microbial DNA isolation kit, as per manufacturers instructions.

***Library preparation and genome sequencing:*** Isolate DNA was sequenced at the Australian Centre for Ecogenomics (ACE) at UQ. DNA was quantitated using Qubit and libraries prepared using Nextera XT library prep (Illumina) with Nextera V2 Indexes, as per manufacturer's instructions. Resulting libraries were quantitated with qPCR, pooled and loaded onto 1/100th of a flow cell per sample and sequenced on the NextSeq (Illumina) using a 2 x 150bp High Output kit. 1.6 million paired end reads from each isolate were used in subsequent bioinformatics analyses.

***QC**:* Raw reads obtained from ACE were initially checked for base pair quality using FastQC (www.bioinformatics.babraham.ac.uk/projects/). Raw reads were then analysed using Kraken[8] to check for contamination. Sample **MS8414**, initially thought to be a CRAB, was determined to be a *K. pnuemoniae* according to the Kraken analysis**.** The original CRAB isolate(s) from this patient ([redacted]) will be retrieved from storage and re-sequenced. Samples **MS8417** and **MS8423** (Both *K. pneumoniae*) were found to be contaminated with reads matching to *A. baumannii* (approximately 3% and 30% contamination respectively) and so were not included in the current data analysis.

### *Bioinformatic analysis*

***CRAB**:* Raw reads obtained from ACE were analysed using Nullarbor[9] to determine sequence type (ST), antibiotic resistance gene profile, phylogeny and core SNP distances using the reference *A. baumannii* Global Clone (GC) 2 strain 1656-2 (GI:384129960). Antibiotic resistance gene content was further checked using a read-mapping tool (srst2) against the ARG-ANNOT database [10]. Multi-locus sequencing typing (MLST) was also performed using srst2 against the *A. baumannii* Oxford MLST scheme (pubmlst.org).

***ESBL-K. pneumoniae**:* Raw reads obtained from ACE were analysed using Nullarbor [9] to determine sequence type (ST), antibiotic resistance gene profile, phylogeny and core SNP distances using the reference *Klebsiella pneumoniae subsp. pneumoniae* MGH 78578 (GI:150953431).

**This report is for research use only** – methods described here are not NATA accredited.

***P. aeruginosa***: Raw reads for our *P. aeruginosa* isolate were analysed directly to determine antibiotic resistance gene content using srst2 against the ARG-ANNOT database.

**TIMELINE:** DNA preparation was carried out on 11th and 15th June following acquisition of isolates (4 hours) and delivered to ACE sequencing facility, UQ (http://ecogenomic.org/services.html) for library preparation on Thursday 16th June (4 hours). Sequencing on Illumina NextSeq began Monday 20th (30 hours). Raw sequence data was delivered to the Beatson Group Wednesday 22nd June 2016 for bioinformatics analysis and reporting (8 hours).

**Bioinformatic analysis:** Leah W Roberts, Dr Brian Forde.
**DNA preparation:** Leah W Roberts (Advised by Dr Minh-Duy Phan, Mark Schembri Lab group)
**Report preparation**: Patrick Harris, Leah W Roberts, Dr Brian Forde
**For further information about these results contact lab head A/Prof Scott Beatson (s.beatson@uq.edu.au)**

**REFERENCES:**


1. Kamolvit W, Sidjabat HE, Paterson DL. Molecular Epidemiology and Mechanisms of Carbapenem Resistance of Acinetobacter spp. in Asia and Oceania. *Microb Drug Resist* 2015; **21**(4): 424-34.
2. Peleg AY, Seifert H, Paterson DL. Acinetobacter baumannii: emergence of a successful pathogen. *Clin Microbiol Rev* 2008; **21**(3): 538-82.
3. Evans BA, Amyes SG. OXA beta-lactamases. *Clin Microbiol Rev* 2014; **27**(2): 241-63.
4. Figueiredo S, Poirel L, Seifert H, Mugnier P, Benhamou D, Nordmann P. OXA-134, a naturally occurring carbapenem-hydrolyzing class D beta-lactamase from Acinetobacter lwoffii. *Antimicrob Agents Chemother* 2010; **54**(12): 5372-5.
5. Sidjabat HE, Townell N, Nimmo GR, et al. Dominance of IMP-4-producing enterobacter cloacae among carbapenemase-producing Enterobacteriaceae in Australia. *Antimicrob Agents Chemother* 2015; **59**(7): 4059-66.
6. Munoz-Price LS, Weinstein RA. Acinetobacter infection. *N Engl J Med* 2008; **358**(12): 1271-81.
7. Girlich D, Naas T, Nordmann P. Biochemical characterization of the naturally occurring oxacillinase OXA-50 of Pseudomonas aeruginosa. *Antimicrob Agents Chemother* 2004; **48**(6): 2043-8.
8. Wood DE, Salzberg SL. Kraken: ultrafast metagenomic sequence classification using exact alignments. *Genome Biol* 2014; **15**(3): R46.
9. Seemann T. Nullarbor v 0.8-dev. 2015. https://github.com/tseemann/nullarbor2016.
10. Gupta SK, Padmanabhan BR, Diene SM, et al. ARG-ANNOT, a new bioinformatic tool to discover antibiotic resistance genes in bacterial genomes. *Antimicrob Agents Chemother* 2014; **58**(1): 212-20.


**This report is for research use only** – methods described here are not NATA accredited.

**School of Chemistry and Molecular Biosciences**
A/Prof Scott Beatson
Head of Microbial Genomics Group
Australian Infectious Diseases Research Centre
Australian Centre for Ecogenomics



August 29th, 2016

**Genome sequencing of Carbapenem-resistant *Acinetobacter baumannii* (CRAB) and companion isolates from RBWH – Batch 4 Summary Report (v2)**

## PREVIOUS REPORTS

To date we have received 4 batches of isolates for sequencing and analysis surrounding the CRAB outbreak at RBWH, totaling 58 isolates (30 CRAB, 2 carbapenem-sensitive *A. baumannii*, 8 ESBL+ *Klebsiella pneumoniae*, 1 carbapenem-resistant *K. pneumoniae,* 1 non-ESBL *K. pneumoniae*, 1 carbapenem resistant *K. pneumoniae*, 7 meropenem-resistant *Pseudomonas aeruginosa*, 5 *Serratia marcescens,* 1 carbapenem-sensitive *Enterobacter cloacae* and 2 carbapenem-resistant *E. cloacae*). We also obtained 15 historical CRAB isolates from between 2000 – 2016 (3 from March/May 2016, 2 from 2015, 10 from between 2000-2006). Analysis and results for these isolate are available as separate reports.

## CURRENT REPORT

This report focuses on the final analysis (batch 4) of 10 new isolates related to the RBWH CRAB outbreak. Isolates were received on the 18th of August and include 1 *S. marcescens*, 7 CRAB and 1 ESBL+ *K. pneumoniae* (Table 1). We also received a borderline carbapenem-resistant *A. baumannii* isolate from a separate location. Sequencing was undertaken at the Australian Centre for Ecogenomics (ACE) on the 22nd August and data was received Wednesday 24th August.

## KEY RESULTS

- The new *S. marcescens* isolate from patient 13 ([redacted]), who was previously colonized with CRAB, appears unrelated to the *S. marcescens* isolated from the index patient ([redacted]) (Figure 1).
- The borderline carbapenem-resistant *A. baumannii* is sequence type (ST) 1122, and unlikely to be related to the current RBWH outbreak CRAB isolates (ST1050). It is also unrelated to the previously isolated carbapenem-resistant *A. baumannii*, differing by roughly 40,000 single nucleotide polymorphisms (SNPs) (Figure 2).
- The remaining CRAB isolates (n=7) were all found to be ST1050 and differ by less than 13 core SNPs (Figure 3), indicating further transmission within the ICU ward.
- The additional ESBL+ *K. pneumoniae* isolated from a new patient (patient 19, [redacted]) was found to be ST515, which is the same as all previously isolated *K. pneumoniae* from this outbreak. It was also found to have an identical antibiotic resistance gene profile and differed by only 2 additional SNPs compared to isolates taken from the index patient ([redacted]) and patient 6 ([redacted]) (Figure 4). This represents further ongoing transmission of *K. pneumoniae* within the ICU ward.

**For an overview of the complete transmission pathway/patient network, see figures 5 and 6 and the end of this document.**

**This report is for research use only** – methods described here are not NATA accredited.

**Table 1: Batch 4 Isolates:** Isolates were received on the 18th August 2016. Genomic DNA was prepared on the 19th August and delivered to the Australian Centre for Ecogenomics (ACE) on the same day. Illumina NextSeq sequencing began on Monday the 22nd August. Raw sequencing data was received on Wednesday the 24th August.

| MS number | Lab number | MRN | Micro Code | Initials | Species | Site | Date | Ward | Comments |
|---|---|---|---|---|---|---|---|---|---|
| MS14424 | [redacted] | [redacted] | [redacted] | PT | *Serratia marcescens* (carb-S) | Rectal swab | 18/07/16 | [redacted] | New - looks different (pigemented) |
| MS14425 | [redacted] | [redacted] | [redacted] | AG | *A. baumannii* (mero MIC=1) | Perianal swab | 26/07/16 | [redacted] | Unlikely to be related |
| MS14426 | [redacted] | [redacted] | [redacted] | LL | CR-A. baum | Urine | 28/07/16 | [redacted] | New |
| MS14427 | [redacted] | [redacted] | [redacted] | PT | CR-A. baum | ETA | 2/08/16 | [redacted] | Previous +ve CRAB |
| MS14428 | [redacted] | [redacted] | [redacted] | SA | CR-A. baum | Swab buttock | 1/08/16 | [redacted] | New |
| MS14429 | [redacted] | [redacted] | [redacted] | JS | CR-A. baum | Swab buttock | 1/08/16 | [redacted] | New |
| MS14430 | [redacted] | [redacted] | [redacted] | HB | CR-A. baum | Blood | 16/05/16 | [redacted] | Previous Serratia mixed isolate |
| MS14431 | [redacted] | [redacted] | [redacted] | HB | CR-A. baum | Blood | 7/05/16 | [redacted] | Index isolate |
| MS14432 | [redacted] | [redacted] | [redacted] | SM | CR-A. baum | Wound swab | 8/08/16 | [redacted] | New |
| MS14433 | [redacted] | [redacted] | [redacted] | SA | *K. pneumoniae* (ESBL) | Rectal swab | 8/08/16 | [redacted] | New |

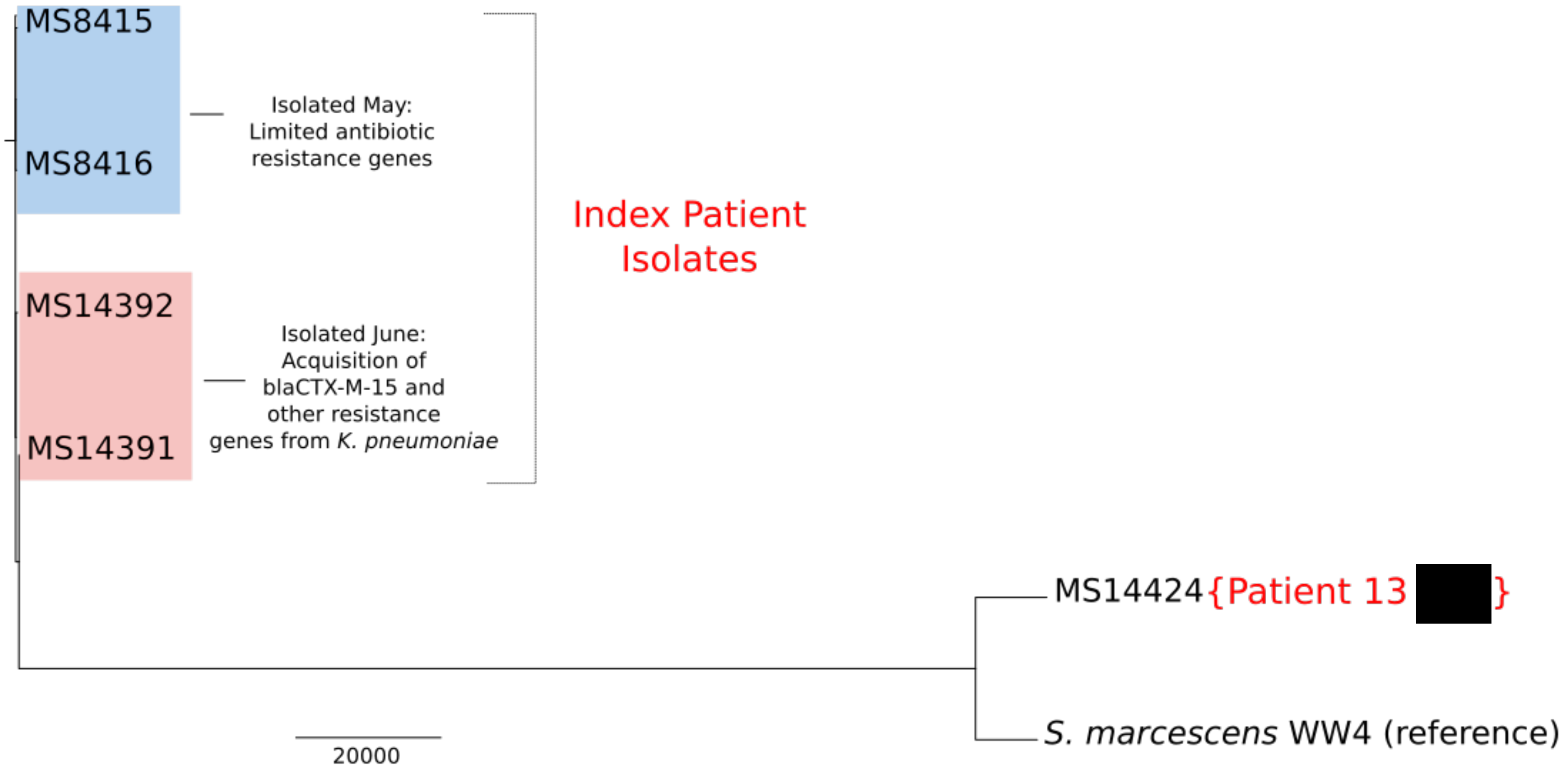


**Figure 1: New *S. marcescens* isolate from patient 13 (PT) is unrelated to 4 isolates previously from index patient ([redacted]):** Branch lengths represent core SNP differences (172993 SNPs overall).

**This report is for research use only** – methods described here are not NATA accredited.

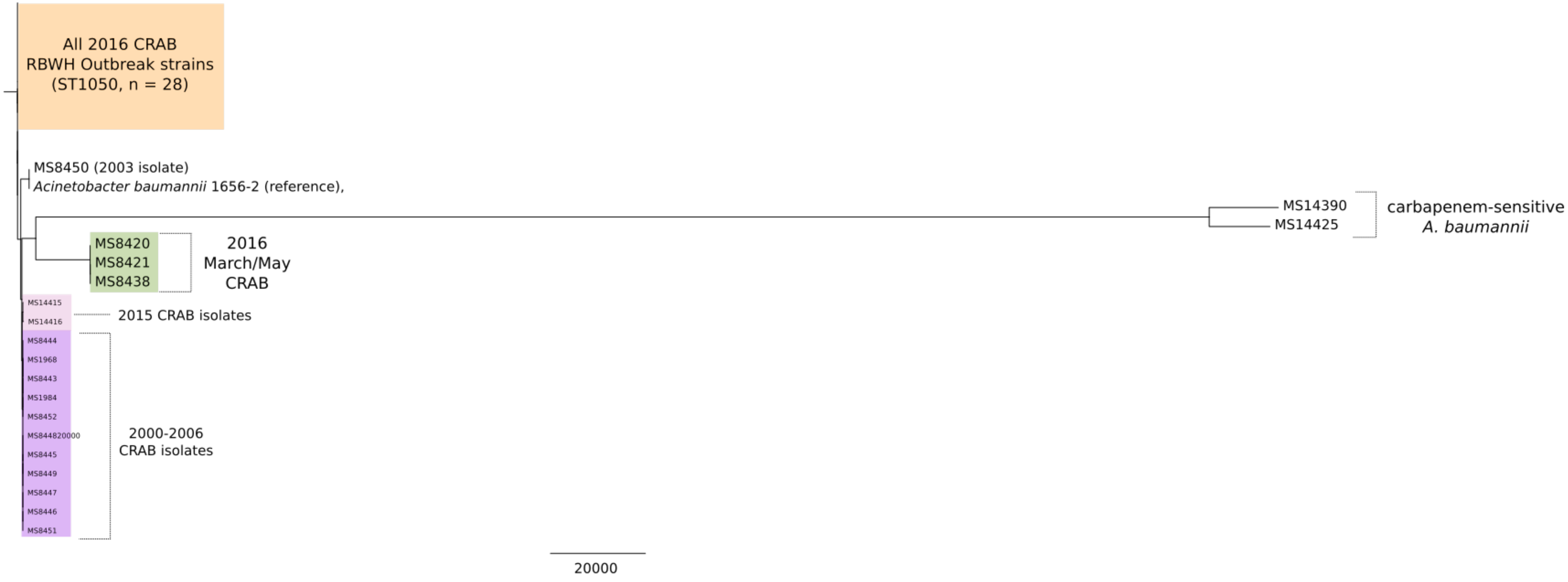


**Figure 2: Summary of relationship between CRAB isolates analysed over the course of the RBWH 2016 June/July CRAB outbreak:** branch lengths represent core SNP differences (186421 SNPs overall)

**This report is for research use only** – methods described here are not NATA accredited.

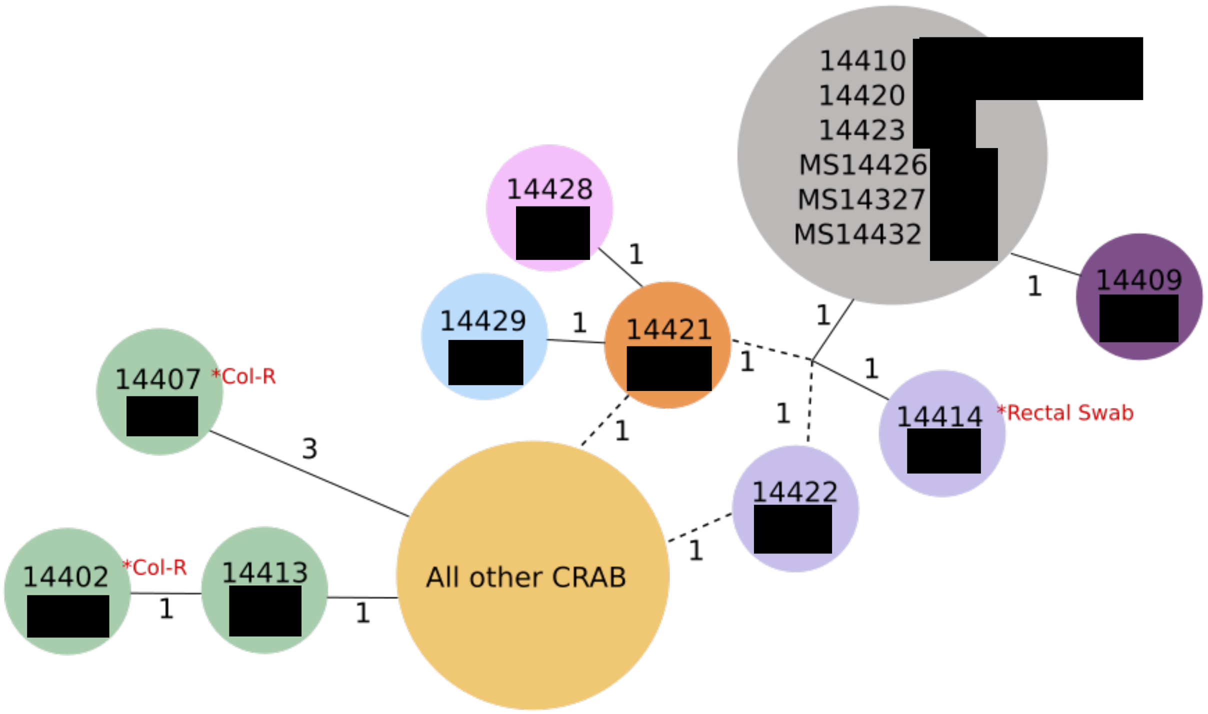


**Figure 3: All ST1050 CRAB isolates from current outbreak differ by less than 13 core SNPs:** Isolates within the same circles are identical are the core SNP level. Solid lines between circles represent accumulating SNPs (exact SNP distance provided). Dotted lines represent two equally likely SNP pathways.

**This report is for research use only** – methods described here are not NATA accredited.

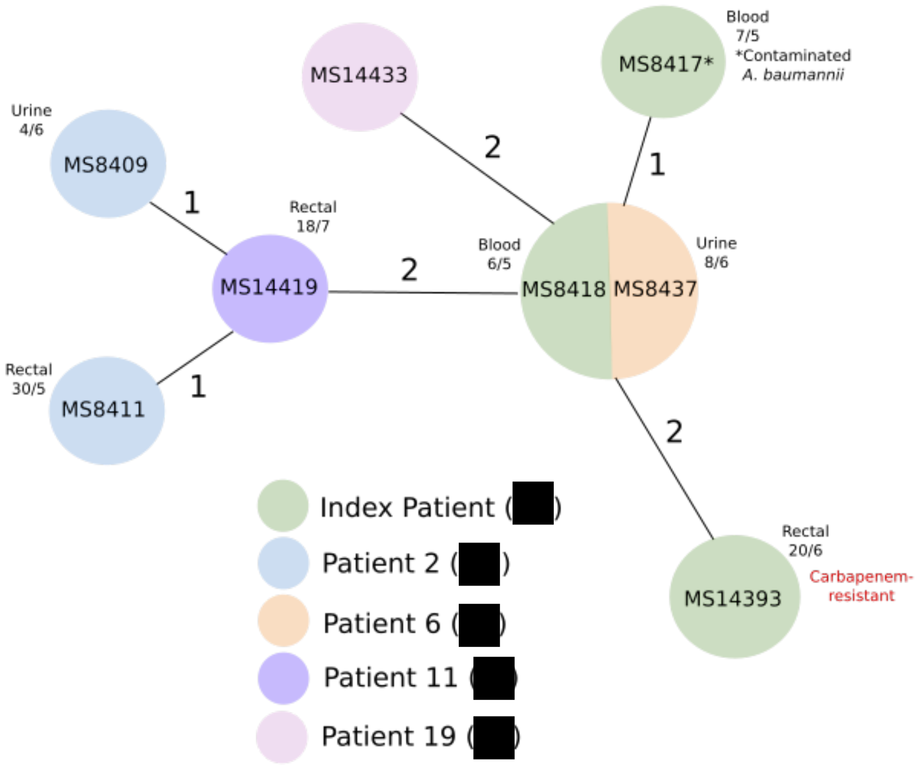


**Figure 4: Ongoing transmission of *K. pneumoniae* in the RBWH ICU ward:** Isolates within the same circle are identical at the core SNP level. Solid lines represent accumulation of SNPs between isolates (specific SNP distances given in figure). The additional *K. pneumoniae* (MS14433, pink) isolate was found to be two core SNPs different to isolates taken from the index patient ([redacted]) and patient 6 ([redacted]). As patients [redacted] and [redacted] had identical *K. pneumoniae* infections, we cannot determine where patient SA has most likely acquired the infection using genomics alone.

**This report is for research use only** – methods described here are not NATA accredited.

**Overall isolate transmission network (Figure 5):**

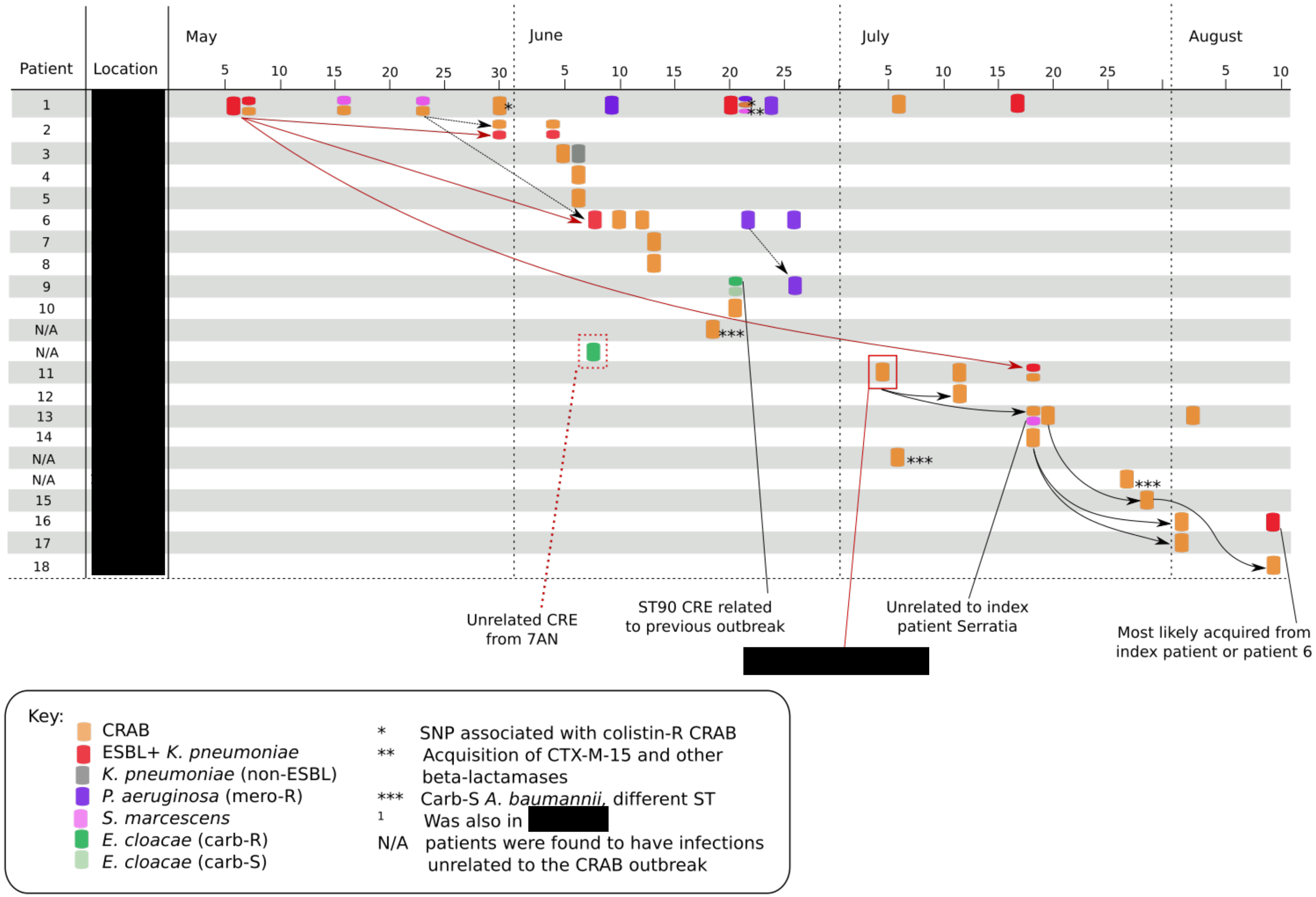


**This report is for research use only** – methods described here are not NATA accredited.

**Overall patient network (Figure 6):**

**Figure 1:**

Each circle represents one patient, where the size of the circle is proportional to the number of isolates from that patient. Lines with arrows represent known transmissions from one patient to other patients. Transmissions where the directionality is unknown are represented with lines only. Arrowed lines that circle back to a single patient represent isolates that were patient specific and not transmitted.

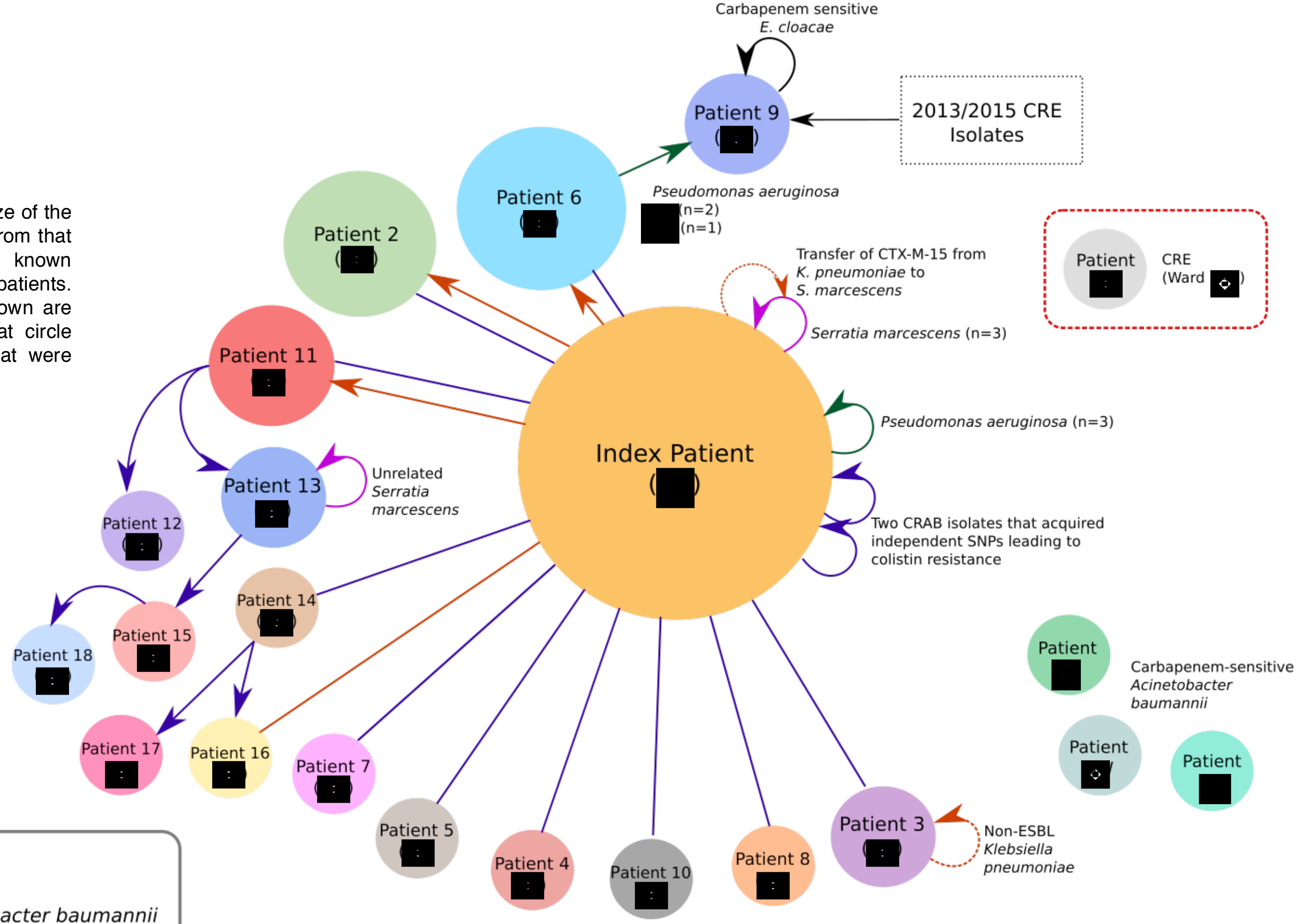


Key:

- Carbapenem-resistant *Acinetobacter baumannii*
- ESBL *Klebsiella pneumoniae*
- *Pseudomonas aeruginosa*
- *Serratia marcescens*

**This report is for research use only** – methods described here are not NATA accredited.

**Methods:**

***Bacterial culture**:* Isolates were delivered to the Schembri Lab on the 18th August 2016. Bacterial culture from each plate was scraped off and resuspended in 5ml LB broth. Pellet from 1.8 ml of this resuspension was then used for genomic DNA extraction using MoBio UltraClean® Microbial DNA isolation kit, as per manufacturers instructions.

***Library preparation and genome sequencing:*** Isolate DNA was sequenced at the Australian Centre for Ecogenomics (ACE) at UQ. DNA was quantitated using Qubit and libraries prepared using Nextera XT library prep (Illumina) with Nextera XT indexes, as per manufacturer's instructions. Resulting libraries were quantitated with Tapestation, pooled and loaded onto 1/200th of a flow cell per sample and sequenced on the NextSeq500 (Illumina) using a 2 x 150bp High Output V2 kit. Approximately 2 million paired end reads from each isolate were used in subsequent bioinformatics analyses.

***QC**:* Raw reads obtained from ACE were initially checked for base pair quality using FastQC (www.bioinformatics.babraham.ac.uk/projects/). Reads were determined the be poor quality at either end, and were subsequently hard trimmed to 100 bp using Nesoni clip (10 bp from start, 40 bp from end) (https://github.com/Victorian-Bioinformatics-Consortium/nesoni). Raw reads were then analysed using Kraken (https://ccb.jhu.edu/software/kraken/) to check for contamination.

***CRAB**:* Raw reads obtained from ACE were analysed using Nullarbor (https://github.com/tseemann/nullarbor) to determine sequence type (ST), antibiotic resistance gene profile, phylogeny and core SNP distances using the reference *A. baumannii* Global Clone (GC) 2 strain 1656-2 (GI:384129960). ST1050 CRAB isolates were then analysed using Nesoni to determine core SNP distances between isolates.

***ESBL-K. pneumoniae**:* Raw reads obtained from ACE were analysed using Nullarbor to determine sequence type (ST), antibiotic resistance gene profile, phylogeny and core SNP distances using the reference *Klebsiella pneumoniae subsp. pneumoniae* MGH 78578 (GI:150953431). The new *K. pneumoniae* isolate was then analysed using Nesoni to determine core SNP distances between all ESBL *K. pneumoniae* in this outbreak.

***Serratia marcescens:*** Raw reads obtained from ACE were analysed using Nullarbor to determine sequence type (ST), antibiotic resistance gene profile, phylogeny and core SNP distances using the reference *Serratia marcescens* WW4 (GI:448239774).

**TIMELINE:** DNA preparation was carried out on the 19th August 206 (6 hours) and delivered to the ACE sequencing facility, UQ (http://ecogenomic.org/services.html) for library preparation on the same day (4 hours). Sequencing on Illumina NextSeq began Monday 22nd (30 hours). Raw sequence data was delivered to the Beatson Group Wednesday 24th June 2016 for bioinformatics analysis and reporting (8 hours).

**Bioinformatic analysis:** Leah W Roberts
**DNA preparation:** Leah W Roberts
**Report preparation**: Leah W Roberts
**For further information about these results contact lab head A/Prof Scott Beatson (s.beatson@uq.edu.au)**

**This report is for research use only** – methods described here are not NATA accredited.
